# CaMKIIβ deregulation contributes to neuromuscular junction destabilization in Myotonic Dystrophy type I

**DOI:** 10.1186/s13395-024-00345-3

**Published:** 2024-05-21

**Authors:** Denis Falcetta, Sandrine Quirim, Ilaria Cocchiararo, Florent Chabry, Marine Théodore, Adeline Stiefvater, Shuo Lin, Lionel Tintignac, Robert Ivanek, Jochen Kinter, Markus A. Rüegg, Michael Sinnreich, Perrine Castets

**Affiliations:** 1 Department of Cell Physiology and Metabolism, Faculty of Medicine, University of Geneva, 1 rue Michel Servet, Geneva, CH-1211 Switzerland; 2 Neuromuscular Research Group, Departments of Neurology and Biomedicine, University and University Hospital Basel, Klingelbergstrasse 50/70, Basel, CH-4056 Switzerland; 3 Biozentrum, University of Basel, Spitalstrasse 41, Basel, CH-4056 Switzerland; 4 Department of Biomedicine, University Hospital and University of Basel, Hebelstrasse 20, Basel, CH-4053 Switzerland; 5 Swiss Institute of Bioinformatics, Hebelstrasse 20, Basel, CH-4053 Switzerland

**Keywords:** Myotonic dystrophy, Neuromuscular junctions, CaMKII, Synaptic genes, Fibre type

## Abstract

**Background:**

Myotonic Dystrophy type I (DM1) is the most common muscular dystrophy in adults. Previous reports have highlighted that neuromuscular junctions (NMJs) deteriorate in skeletal muscle from DM1 patients and mouse models thereof. However, the underlying pathomechanisms and their contribution to muscle dysfunction remain unknown.

**Methods:**

We compared changes in NMJs and activity-dependent signalling pathways in *HSA*^*LR*^ and *Mbnl1*^*ΔE3/ΔE3*^ mice, two established mouse models of DM1.

**Results:**

Muscle from DM1 mouse models showed major deregulation of calcium/calmodulin-dependent protein kinases II (CaMKIIs), which are key activity sensors regulating synaptic gene expression and acetylcholine receptor (AChR) recycling at the NMJ. Both mouse models exhibited increased fragmentation of the endplate, which preceded muscle degeneration. Endplate fragmentation was not accompanied by changes in AChR turnover at the NMJ. However, the expression of synaptic genes was up-regulated in mutant innervated muscle, together with an abnormal accumulation of histone deacetylase 4 (HDAC4), a known target of CaMKII. Interestingly, denervation-induced increase in synaptic gene expression and AChR turnover was hampered in DM1 muscle. Importantly, CaMKIIβ/βM overexpression normalized endplate fragmentation and synaptic gene expression in innervated *Mbnl1*^*ΔE3/ΔE3*^ muscle, but it did not restore denervation-induced synaptic gene up-regulation.

**Conclusions:**

Our results indicate that CaMKIIβ-dependent and -independent mechanisms perturb synaptic gene regulation and muscle response to denervation in DM1 mouse models. Changes in these signalling pathways may contribute to NMJ destabilization and muscle dysfunction in DM1 patients.

**Supplementary Information:**

The online version contains supplementary material available at 10.1186/s13395-024-00345-3.

## Background

Myotonic Dystrophy type I (DM1) is a multisystemic disorder caused by expanded CTG triplet repeats in the 3’UTR of the *DMPK* (*Dystrophia Myotonica Protein Kinase*) gene that leads to muscle wasting, weakness and inability to relax (myotonia). Accumulation of toxic transcripts containing the expanded CUG repeats leads to the nuclear sequestration of splicing factors. The consecutive mis-splicing of specific genes is determinant in the pathogenesis of DM1-associated muscle alterations [1, 2]. For example, mis-splicing of the *CLCN1* gene, which encodes the chloride channel ClC-1, is implicated in myotonia development. Mis-splicing of several genes encoding proteins of Ca^2+^-associated signalling pathways has also been shown to contribute to muscle dysfunction. In particular, previous reports revealed that mis-splicing of *CAMK2* genes, encoding Ca^2+^/calmodulin-dependent protein kinases II (CaMKIIs), is a hallmark of DM1 [3–5]. However, the functional consequences of CAMKII deregulation in DM1 muscle and its contribution to DM1 pathogenesis have not been explored.

CaMKIIs are important for the maintenance of neuromuscular junctions (NMJs), which are the synapses connecting motor neurons to muscle fibres. Especially, CaMKIIs enhance the recycling of acetylcholine receptors (AChRs) upon their internalization in the sub-synaptic region of muscle fibres (i.e., the endplate) [6]. Moreover, CaMKIIs indirectly repress the expression of synaptic genes in non-synaptic regions of innervated muscle, by inhibiting the myogenic transcription factor myogenin and the histone deacetylase 4 (HDAC4) [7–9]. Hence, deregulation of the CaMKII signalling pathway may affect NMJ maintenance, by perturbing the expression and the dynamics of synaptic proteins.

Early studies on DM1 muscle biopsies pointed to NMJ-associated abnormalities, such as a reduced number of presynaptic vesicles, enlarged endplates or angular muscle fibres [10–13]. NMJ alterations have also been reported in *DMSXL* mice and in mice deficient for Muscleblind-like 1/2 (Mbnl1/2), two mouse models of DM1, as well as in *C. elegans* DM1 mutants [14, 15]. The lack of denervation markers (e.g., non-junctional AChR clusters) and the absence of massive motor neuron loss rejected the hypothesis that spontaneous denervation is part of DM1 pathogenesis [16, 17]. However, the contribution of neuronal deregulations to NMJ defects has recently been proposed from human motoneuron / muscle cells co-culture [18]. Nuclear foci, characteristic of DM1-associated accumulation of toxic RNA, were detected at the endplate and may alter the expression of synaptic genes [19]. Consistently, mis-splicing of some synaptic genes has been reported in muscle cells from DM1 patients [20]. Hence, NMJ deterioration in DM1 is likely to contribute to muscle dysfunction, but the underlying pathomechanisms remain unknown.

Here, we analysed changes in activity-dependent signalling pathways in *HSA*^*LR*^ and *Mbnl1*^*ΔE3/ΔE3*^ mice, two established DM1 mouse models. Both mouse models displayed increased fragmentation of the endplate, which preceded muscle alterations. *HSA*^*LR*^ and *Mbnl1*^*ΔE3/ΔE3*^ mice also showed major deregulation of CaMKIIβ signalling in muscle, which contributed to increase synaptic gene expression and endplate fragmentation in innervated conditions. Interestingly, the muscle response to denervation was hampered in *Mbnl1*^*ΔE3/ΔE3*^ mice, independently from CaMKIIβ/βM deficiency. Hence, perturbations in activity-dependent signalling pathways may contribute to NMJ destabilization and muscle dysfunction in DM1 patients.

## Methods

### Mice

Homozygous mice of the mouse line LR20b carrying about 250 (CTG) repeats within the *HSA* transgene (*HSA*^*LR*^) were obtained from Thornton and colleagues (University of Rochester Medical Centre, Rochester, New York, USA) [21]. Mice of the corresponding background strain (FVB/N) were used as controls. Mice were genotyped for the *HSA*^*LR*^ transgene by quantifying human *ACTA1* levels normalized to endogenous actin (mouse *Acta1*) in genomic DNA. Mice from the *Mbnl1ΔE3* line were obtained from Swanson and colleagues (College of Medicine, University of Florida, Gainesville, Florida, USA) [22]. *Mbnl1*^*+/+*^ littermates from the corresponding background strain (C57BL/6) were used as controls. Mice from the *Mbnl1ΔE3* line were genotyped for exon 3 depletion at the *Mbnl1* locus. Mice were maintained in a conventional specific-pathogen-free facility with a fixed light cycle (23 °C, 12 h dark-light cycle). For the AAV study, anterior hindlimb compartments of control and mutant mice were injected with adeno-associated virus serotype 9 (AAV9; GeneCopoeia) carrying either the transgene for CaMKIIβ (AA09-Mm33884-AV01-A00-GS), CaMKIIβM (AA09-Mm44818-AV01-A00-GS) or GFP (AA09-NEG-AV07-A00) at a dose of 8 × 10^10^ viral particles per compartment. Sciatic nerve cut was conducted as described previously [23]. All animal studies were performed in accordance with the European Union guidelines for animal care and approved by the Veterinary Office of the Cantons of Basel city (application number 2601) and Geneva (application number GE220/GE227).

### Muscle force and relaxation

In vitro force measurement of EDL muscle and late relaxation time evaluation were conducted as previously described [24, 25].

### Western blotting

Muscles powdered in liquid nitrogen were lysed in cold RIPA + buffer (50 mM Tris-HCl pH 8, 150 mM NaCl, 1% NP-40, 0.5% sodium deoxycholate, 0.1% SDS, 1% Triton X, 10% glycerol, phosphatase and protease inhibitors). Subcellular fractionation was done according to Dimauro et al. (2012) [26]. Following dosage (BCA Protein Assay, Pierce), proteins were separated on SDS-polyacrylamide gels and transferred to nitrocellulose membrane. Blots were blocked in TBS, 3% BSA, 0.1% Tween-20, and incubated overnight at 4 °C with primary antibodies, then for 2 h with HRP-labelled secondary antibodies. Immunoreactivity was detected using the Western blot chemiluminescent substrate LumiGLO (Seracare) and exposed to Super RX-N films (Fujifilm) or revealed with iBright™ Imaging System (ThermoFisher). Protein expression was normalized to α-actinin or GAPDH, or to total protein levels of the corresponding phosphorylated form. The list of antibodies used is provided in Supplementary Materials.

### Polymerase chain reaction

Total RNAs were extracted with the RNeasy Mini Kit (Qiagen), reverse transcribed with the SuperScript III First-Strand Synthesis System (Invitrogen), and amplified with the Power SYBR Green Master Mix (Applied Biosystems) or the Hot FirePol EvaGreen qPCR Mix (Solis BioDyne). Expression of specific spliced or pan transcripts was analysed by end-point PCR and electrophoresis, or by quantitative PCR with the Step-One software and normalization to *Tbp* expression. The list of primers used is provided in Table S1.

### Histology and immunofluorescence

Muscles were frozen in liquid nitrogen-cooled isopentane. Eight-micrometre muscle sections were stained with H&E and observed with an upright microscope (Olympus). For immunostaining, sections were fixed with 4% paraformaldehyde (PFA) or kept unfixed, then blocked in PBS, 3% BSA, incubated sequentially with primary and secondary fluorescent antibodies (Invitrogen, Jackson ImmunoResearch), mounted with Vectashield medium (Vector), and observed with Leica or Zeiss fluorescent microscopes. Quantification of fibre type and size was done as previously reported [27].

### Muscle bundle staining

To analyse NMJ organization, muscles were bathed ex vivo with α-bungarotoxin(Btx)-Alexa555 (2 µg/ml - Invitrogen) for 30 min, before being washed and fixed with 4% PFA. Muscle bundles were cut, permeabilized in PBS, 1% Triton-X100, and blocked in PBS, 1% BSA, 0.1% Triton-X100. Bundles were then successively incubated with primary antibodies against neurofilament and synaptophysin (to stain pre-synaptic compartment), and the corresponding secondary antibodies (Invitrogen, Jackson ImmunoResearch). Images were recorded using Leica and Zeiss confocal microscopes. 3D reconstructed images of endplates, based on Btx staining, were obtained with Imaris 10.1.1 software. The number of AChR fragments per endplate were counted manually in a double-blind manner. Volume and projected area of Btx-stained AChRs per endplate were measured with ImageJ/Fiji software.

### AChR turnover

AChR turnover was assessed by injecting Btx-Alexa647 and -Alexa555 (25 pmoles - Invitrogen) into TA/EDL muscles at days 1 and 10, respectively (5 and 14 days after nerve cut when combined with denervation), as previously described [23]. For turnover quantification, images were recorded using Leica or Zeiss confocal microscopes. Pixel dominance (old vs. new receptors) was calculated using Fiji and MATLAB software. The assays could be applied to TA and EDL muscles, but not to the *gastrocnemius* muscle because of its size and heterogeneity.

### Statistics

Quantitative data are displayed as mean ± SEM of independent samples, with n (number of individual experiments) ≥ 3. The statistical analysis of values was performed using unpaired Student’s t test or two-way ANOVA test with Tukey’s multiple comparisons test correction, with prior log transformation of the data that were not normally distributed (RNA and protein levels, relaxation time). A 0.05 level of confidence was accepted for statistical significance.

## Results

### DM1-associated muscle alterations are similar in *Mbnl1*^*ΔE3/ΔE3*^ and *HSA*^*LR*^ mice

**Fig. 1:**
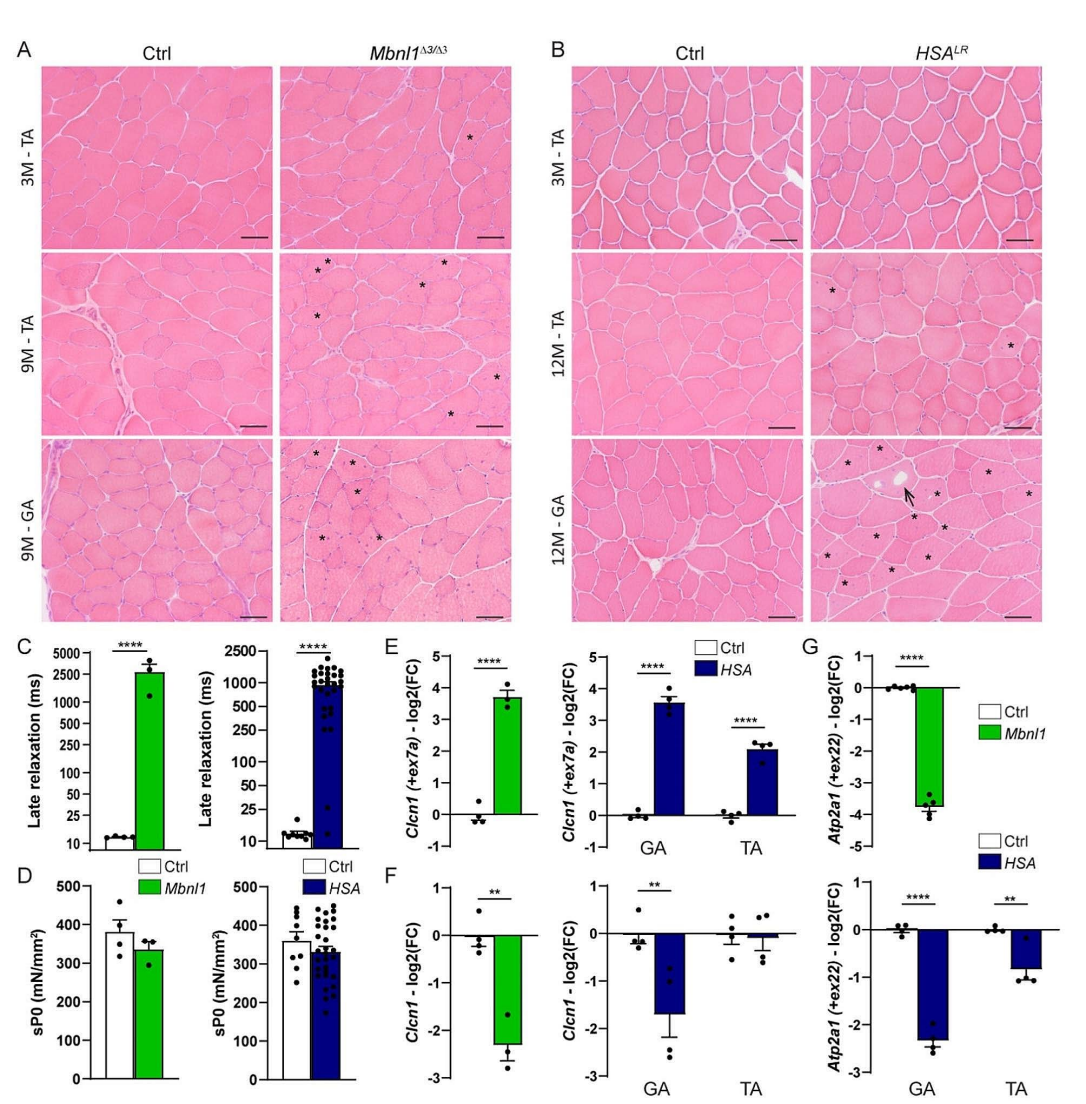
Muscle phenotype in *Mbnl1*^*ΔE3/ΔE3*^ and *HSA*^*LR*^ mice. **A, B** H&E coloration reveals moderate myopathic alterations in TA and *gastrocnemius* (GA) muscles from 3- and 9-month(M)-old *Mbnl1*^*ΔE3/ΔE3*^ (A) and 3- and 12-month(M)-old *HSA*^*LR*^ mice (B). Asterisks and arrows point to internalized nuclei and vacuoles, respectively. Scale bar, 100 μm. **C** Late relaxation time upon stimulation is increased in EDL muscle from 3-month-old *Mbnl1*^*ΔE3/ΔE3*^ and *HSA*^*LR*^ mice, as compared to control mice. *n* = 4 Ctrl / 3 *Mbnl1*^*ΔE3/ΔE3*^; 9 Ctrl / 30 *HSA*^*LR*^. **D** Specific tetanic force (sP0) of EDL muscle is unchanged in 3-month-old *Mbnl1*^*ΔE3/ΔE3*^ (*n* = 4 Ctrl / 3 KO) and *HSA*^*LR*^ (*n* = 9 Ctrl / 30 *HSA*^*LR*^) mice. **E-G** qPCR analysis of *Clcn1* exon7a inclusion (E), *Clcn1* total mRNA levels (F) and *Atp2a1* exon22 inclusion (G) in TA muscle from *Mbnl1*^*ΔE3/ΔE3*^ mice and *gastrocnemius* (GA) or TA muscle from *HSA*^*LR*^ mice. Expression is normalised on total mRNA expression for splice variants (E, G) or on *Tbp* expression (F). Levels are relative to control and expressed as log2(Fold Change). *n* = 4 Ctrl / 3 *Mbnl1*^*ΔE3/ΔE3*^ (E, F); 6 Ctrl / 5 *Mbnl1*^*ΔE3/ΔE3*^ (G); 4 Ctrl / 4 *HSA*^*LR*^. All data represent mean ± SEM. **p < 0.01; ****p < 0.0001; unpaired two-tailed Student’s t test

To evaluate changes in NMJs and activity-dependent pathways in DM1, we selected the *Mbnl1*^*ΔE3/ΔE3*^ and *HSA*^*LR*^ mouse lines, which are two well-established DM1 mouse models. In *Mbnl1*^*ΔE3/ΔE3*^ mice, deletion of *Mbnl1* exon 3 leads to body-wide depletion of the splicing factor MBNL1 [22]. In contrast, *HSA*^*LR*^ mice express the *HSA* transcript (*Human Skeletal Actin*) with long (CTG) repeats only in skeletal muscle, which allowed to unveil cell-autonomous defects [21]. First, we compared the muscle phenotype between the two mouse models. There was no dystrophic sign in muscles from 3-month-old mutant mice (Fig. 1A, B), as previously reported [21, 22]. The dystrophic phenotype remained moderate in 9- and 12-month-old *Mbnl1*^*ΔE3/ΔE3*^ and *HSA*^*LR*^ mice, respectively (Fig. 1A, B). In particular, fibre size variation, increased proportion of fibres with internalized myonuclei, as well as intracellular vacuoles were present in *Mbnl1*^*ΔE3/ΔE3*^*tibialis anterior* (TA) and *gastrocnemius* muscles (Fig. 1A), as well as in *gastrocnemius* muscle from *HSA*^*LR*^ mice (Fig. 1B). In contrast, these alterations were rare in TA muscle from *HSA*^*LR*^ mice (Fig. 1B). This was consistent with previous reports describing differential muscle severity in this mouse model [28], which may pertain to the *HSA* promoter. This heterogeneity between muscles was not observed in *Mbnl1*^*ΔE3/ΔE3*^ mice. Both *Mbnl1*^*ΔE3/ΔE3*^ and *HSA*^*LR*^ mice exhibited a myotonic phenotype, as shown by the increased late relaxation time of *extensor digitorum longus* (EDL) muscle after ex vivo stimulation at 3 months (Fig. 1C and Fig. S1A) and 9/12 months (Fig. S1B) of age. In contrast, specific tetanic muscle force (sP0) was unaffected in 3-month-old mutant mice (Fig. 1D) and only slightly reduced in 9-month-old *Mbnl1*^*ΔE3/ΔE3*^ mice (Fig. S1C).

To evaluate DM1-associated mis-splicing, we next quantified the inclusion of exons 7a and 22 of the *Clcn1* and *Atp2a1* genes, which encode ClC-1 channel and SERCA1 (Sarco/Endoplasmic Reticulum Ca^2+^-ATPase), respectively. The inclusion of *Clcn1* exon 7a was strongly increased in *Mbnl1*^*ΔE3/ΔE3*^ TA muscle (Fig. 1E) and was accompanied by major reduction in total *Clcn1* transcript levels (Fig. 1F). *Clcn1* mis-splicing and down-regulation were similar in *gastrocnemius* muscle from *HSA*^*LR*^ mice (Fig. 1E, F). In contrast, TA muscle from *HSA*^*LR*^ mice showed milder changes in *Clcn1* splicing and no reduction in total *Clcn1* transcript levels (Fig. 1E, F). Similarly, the inclusion of *Atp2a1* exon 22 was abrogated in *Mbnl1*^*ΔE3/ΔE3*^ TA muscle and in *gastrocnemius* muscle from *HSA*^*LR*^ mice, while it was reduced by only half in *HSA*^*LR*^ TA muscle (Fig. 1G). These results confirm that both mouse models have mild muscle alterations, with similar DM1-associated phenotype observed in *Mbnl1*^*ΔE3/ΔE3*^ muscles and *HSA*^*LR*^*gastrocnemius* muscle, and milder changes detected in TA muscle from *HSA*^*LR*^ mice.

### CaMKIIs are strongly deregulated in muscle from DM1 patients and mouse models

**Fig. 2:**
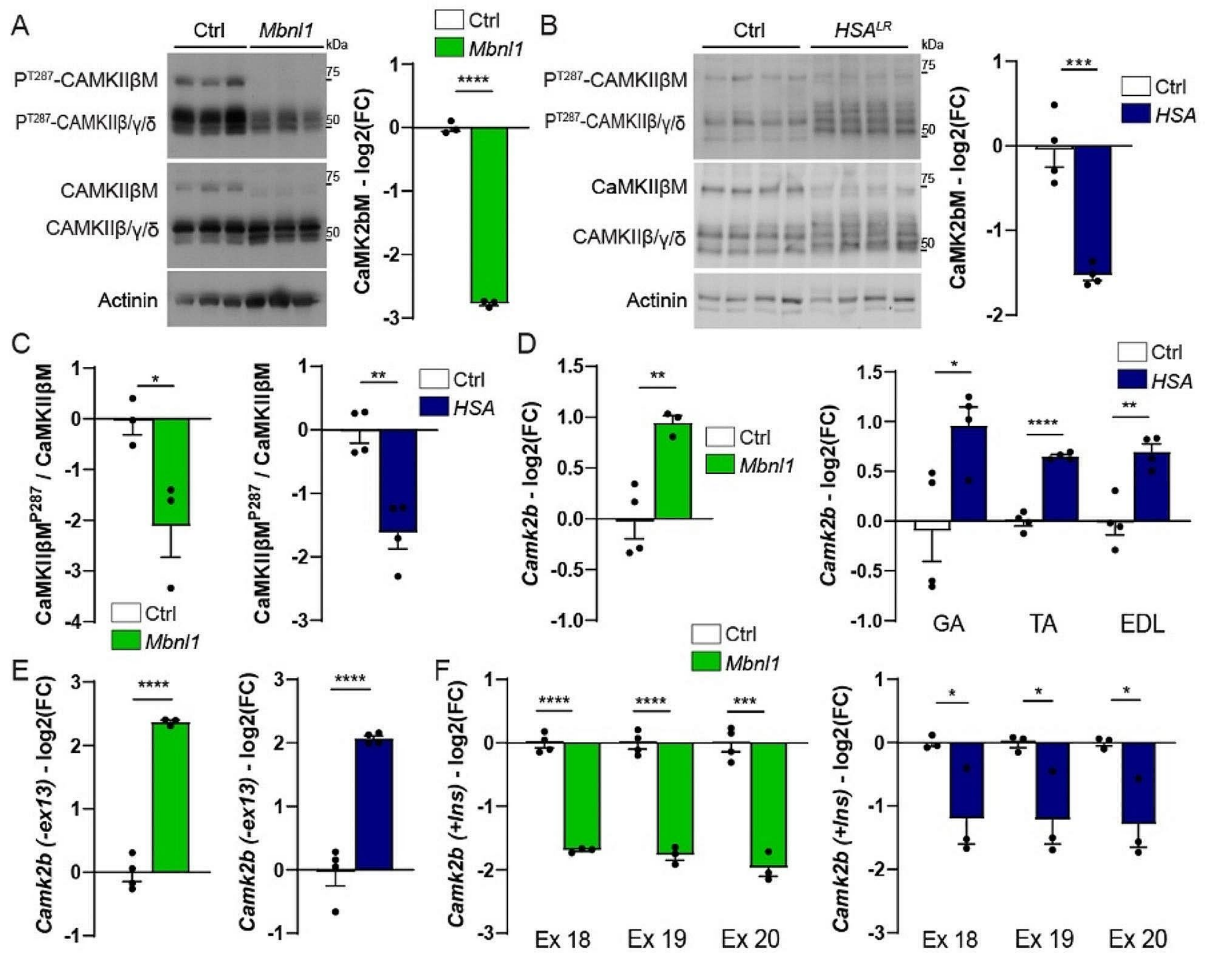
CaMKIIβ deregulation in *Mbnl1*^*ΔE3/ΔE3*^ and *HSA*^*LR*^ muscles. **A-C** Western blot analysis of CaMKII isoforms and quantification of CaMKIIβM levels in TA muscle from 3-month-old *Mbnl1*^*ΔE3/ΔE3*^ mice (A) and in *gastrocnemius* muscle from 3-month-old *HSA*^*LR*^ mice (B). Quantification of CaMKIIβM phosphorylated form in *Mbnl1*^*ΔE3/ΔE3*^ and *HSA*^*LR*^ muscles is shown in C. Protein levels are normalized on α-actinin (A, B) or on total CaMKIIβM (C), relative to control and expressed as log2(Fold Change). *n* = 3 (Ctrl/*Mbnl1*^*ΔE3/ΔE3*^) and 4 (Ctrl/*HSA*^*LR*^) per group. **D-F** Quantitative RT-PCR analysis of total *Camk2b* mRNA levels (D), *Camk2* exon 13 exclusion (E), and *Camk2b* exons 18–20 inclusion (F) in TA muscle from *Mbnl1*^*ΔE3/ΔE3*^ mice and in *gastrocnemius* (GA), TA and EDL muscles from *HSA*^*LR*^ mice. Data are normalized on *Tbp* levels (D) or on total *Camk2b* transcripts (E, F), relative to control and expressed as log2(Fold Change). *n* = 4 Ctrl / 3 *Mbnl1*^*ΔE3/ΔE3*^; 4 Ctrl / 4 *HSA*^*LR*^ (D, E); 3 Ctrl / 3 *HSA*^*LR*^ (F). All data are mean ± SEM; * *p* < 0.05; ** *p* < 0.01; ***p < 0.001; **** *p* < 0.0001; two-tailed unpaired Student’s t-test

Previous reports pointed to mis-splicing of *CAMK2* genes in DM1 tissues [3–5]. However, it remains unclear which CaMKII isoforms are affected in DM1 muscle, and what the consequences of their deregulation are. CaMKIIβ/γ/δ isoforms are encoded by the three genes *CAMK2B, 2G* and *2D*, and are expressed in skeletal muscle. Each gene is expressed as different splice variants. In particular, CaMKIIβ splice variants include CaMKIIβ/βe/β’/βe’ and the muscle-specific variant CaMKIIβM, which arise from the alternative splicing of *CAMK2b* exons 13, 16 and 18–20 (Fig. S2A) [29–31]. Protein levels of CaMKIIβM, which can be distinguished from other CaMKII isoforms based on its size, were strongly reduced in both *Mbnl1*^*ΔE3/ΔE3*^ (Fig. 2A) and *HSA*^*LR*^ (Fig. 2B) muscles. Auto-phosphorylation of CaMKIIβM (phospho-Thr287) decreased as well (Fig. 2C), suggesting reduced CaMKIIβM activity. Notably, additional bands around the size of the other isoforms of CaMKIIβ and of CaMKIIγ/δ suggest the expression of alternative CaMKII isoforms in mutant muscles (Fig. 2A, B).

To characterize splicing events in the *Camk2b, 2d* and *2g* genes in DM1 muscle, we used RNA-seq data that we obtained from *HSA*^*LR*^*gastrocnemius* muscle. We detected significant changes in *Camk2b* transcript, with a major exclusion of exons 13 and 18 to 20 in *HSA*^*LR*^ muscle, compared to control (Fig. S2B, C). There was no change in the splicing of *Camk2b* exon 16 in *HSA*^*LR*^ muscle (Fig. S2B, C). We confirmed the expression of *Camk2b* transcripts lacking exon 13 (corresponding to CaMKIIβe/e’) in *HSA*^*LR*^ muscle by RT-PCR (Fig. S2D). Moreover, the amplicon including exons 18 to 20, which encode the variable inserts of CaMKIIβM, was barely detected in *HSA*^*LR*^ muscle (Fig. S2D). By quantitative RT-PCR, total levels of *Camk2b* transcripts increased in *Mbnl1*^*ΔE3/ΔE3*^ TA muscle, as well as in *HSA*^*LR*^*gastrocnemius*, TA and EDL muscles (Fig. 2D). Using primers spanning *Camk2b* exons 12–14, we confirmed that the expression of *Camk2b* transcripts without exon 13 was more than four times higher in mutant muscles than in controls (Fig. 2E). Conversely, levels of *Camk2b* transcripts with exons 18 to 20 were reduced by half in *Mbnl1*^*ΔE3/ΔE3*^ TA muscle and in *gastrocnemius* muscle from *HSA*^*LR*^ mice (Fig. 2F) compared to controls. Notably, TA and EDL muscles from *HSA*^*LR*^ mice showed similar extent of *Camk2b* mis-splicing compared to *gastrocnemius* muscle (Fig. S2E, F). Interestingly, the mis-splicing of exon 13 and exons 18–20 is also confirmed by RNA-seq data of TA muscle from DM1 patients (Fig. S3) [32]. These results indicate that skeletal muscles from DM1 patients and mouse models shift to the expression of CaMKIIβe isoform (arising from exons 13/18–20 exclusion, with exon 16 inclusion), while CaMKIIβ (+ ex13 + ex16Δex18-20) and CaMKIIβM (+ ex13 + ex16 + ex18-20) are the predominant isoforms expressed in control muscles.

Transcripts encoding CaMKIIγ were also mis-spliced in *HSA*^*LR*^ muscle. These included increased inclusion of exons 13, 15 and 19 in *Camk2g* transcripts in *HSA*^*LR*^ muscle, as seen in RNA-seq reads (Fig. S4A, B) and by RT-PCR (Fig. S4C). Similar *Camk2g* mis-splicing was observed in *Mbnl1*^*ΔE3/ΔE3*^ TA muscle (Fig. S4D). Increased inclusion of *CAMK2G* exon 19 was also observed in TA biopsies from DM1 patients (Fig. S4E) [32]. In contrast, there was no major splicing change detected for *Camk2d* in *HSA*^*LR*^ muscle (Fig. S5A) and *CAMK2D* in DM1 muscle (Fig. S5B) [32]. Taken together, these results show that mis-splicing of some *Camk2* transcripts alters the expression pattern of CaMKIIs in DM1 muscle.

### Endplate fragmentation is not caused by abnormal AChR turnover in *Mbnl1*^*ΔE3/ΔE3*^ and *HSA*^*LR*^ mice

**Fig. 3:**
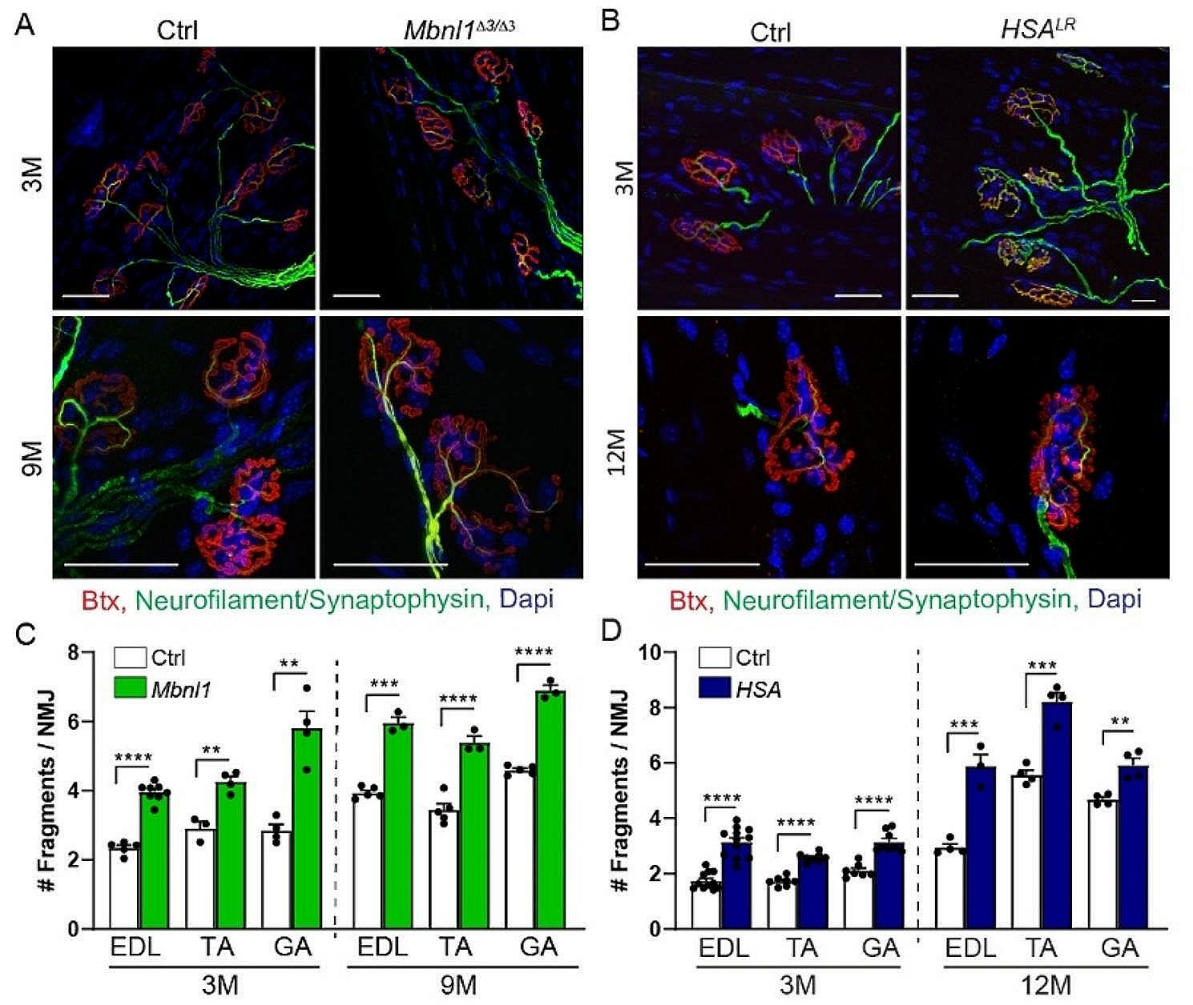
Altered NMJ maintenance in *Mbnl1*^*ΔE3/ΔE3*^ and *HSA*^*LR*^ mice. **A, B** Fluorescent images of NMJ regions stained with α-bungarotoxin (Btx, red), antibodies against neurofilament/synaptophysin (green), and dapi (blue) in EDL muscles from 3- and 9-month-old *Mbnl1*^*ΔE3/ΔE3*^ mice (A) and 3-and 12-month-old *HSA*^*LR*^ mice (B). Scale bar, 50 μm. **C, D** Quantification of the number of fragments per endplate in EDL, TA and *gastrocnemius* (GA) muscles from 3- and 9/12-month-old *Mbnl1*^*ΔE3/ΔE3*^ (C) and *HSA*^*LR*^ (D) mice. *n* = 5/8 (EDL 3 M), 3/4 (TA 3 M), 4/4 (GA 3 M), 5/3 (all muscles 9 M) Ctrl/ *Mbnl1*^*ΔE3/ΔE3*^ (C); 11/12 (EDL 3 M), 7/8 (TA 3 M), 7/8 (GA 3 M), 4/3 (EDL 12 M), 4/4 (TA and GA 12 M) Ctrl/*HSA*^*LR*^ (D), with more than 50 fibres per muscle. Data represent mean ± SEM. **p < 0.01, ***p < 0.001, ****p < 0.0001, unpaired two-tailed Student’s t test

As CaMKIIs are key sensors of neural activity involved in NMJ maintenance, we analysed NMJ structures in EDL, TA and *gastrocnemius* muscles from 3-month-old and 9- or 12-month-old *Mbnl1*^*ΔE3/ΔE3*^ and *HSA*^*LR*^ mice. Pre- and post-synaptic compartments were stained in whole-mount muscle bundles with antibodies against neurofilament/synaptophysin and with α-bungarotoxin (Btx), which binds specifically to AChRs, respectively. The overall organization of the NMJs was preserved in mutant mice (Fig. 3A, B). In particular, we did not observe signs of denervation or abnormal axonal termination in mutant muscles. Acetylcholinesterase staining was also similar in control and mutant mice (Fig. S6A, B). However, the number of AChR fragments per endplate increased in muscles from both 3- and 9-month-old *Mbnl1*^*ΔE3/ΔE3*^ mice, compared to age-matched controls (Fig. 3C and Fig. S6C). A similar increase in endplate fragmentation was observed in *gastrocnemius*, TA and EDL muscles from *HSA*^*LR*^ mice (Fig. 3D and Fig. S6D). Of note, the volume occupied by AChRs was unchanged in *Mbnl1*^*ΔE3/ΔE3*^ and *HSA*^*LR*^ mice (Fig. S6E). There was a slight increase in the projected endplate area in *HSA*^*LR*^ EDL muscle, which may arise from endplate fragmentation (Fig. S6F). As the *HSA*^*LR*^ transgene is specifically expressed in skeletal muscle, these results indicate that post-synaptic perturbations contribute to endplate fragmentation in *HSA*^*LR*^ mice. Moreover, these NMJ alterations were detected as soon as 3 months of age, i.e., before changes in muscle histology, suggesting that they are primary deficit in DM1 mouse models and not a consequence of muscle degeneration/regeneration.

**Fig. 4:**
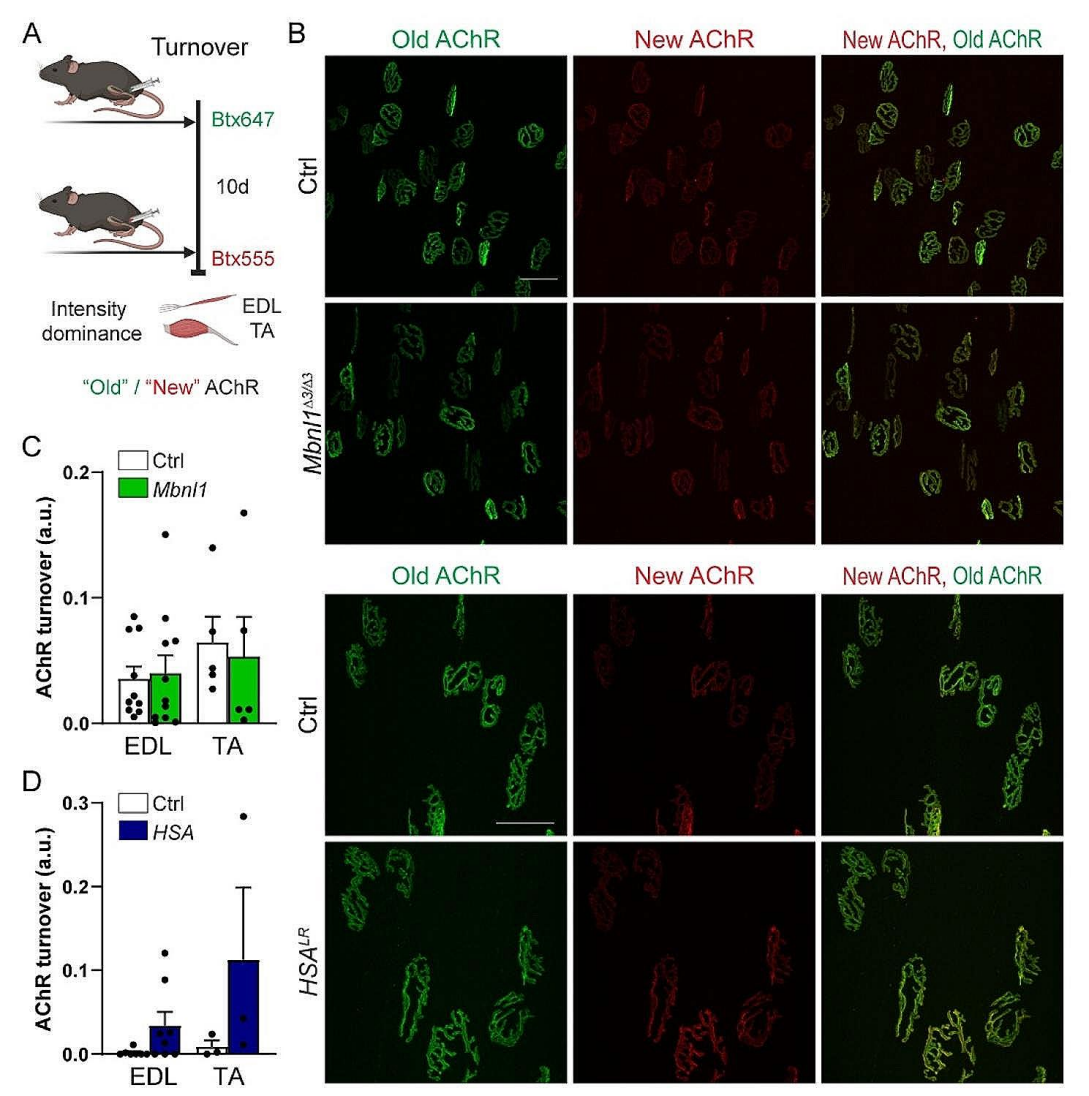
AChR dynamics is not altered in *Mbnl1*^*ΔE3/ΔE3*^ and *HSA*^*LR*^ muscles. **A** Timeline of injections of α-bungarotoxin (Btx) for AChR turnover assay. *d, days.* Created with BioRender.com. **B** Turnover assay in EDL and TA muscles from *Mbnl1*^*ΔE3/ΔE3*^ and *HSA*^*LR*^ mice. Fluorescent images show “old” (green) and “new” (red) AChRs in *Mbnl1*^*ΔE3/ΔE3*^ and *HSA*^*LR*^ muscles. Scale bar, 50 μm. **C, D** AChR turnover in EDL and TA muscles from *Mbnl1*^*ΔE3/ΔE3*^ (C) and *HSA*^*LR*^ (D) mice. *n* = 10/11 (EDL) and 5/5 (TA) Ctrl/*Mbnl1*^*ΔE3/ΔE3*^ (C); 7/8 (EDL) and 3/3 (TA) Ctrl/*HSA*^*LR*^ (D), with more than 22 fibres per muscle. Data are mean ± SEM.

As CaMKIIs have been shown to regulate AChR recycling at the endplate [6], we next evaluated AChR turnover, by labelling “old” and newly formed receptors by two sequential injections of differently labelled Btx (Fig. 4A, B) [23, 33]. AChR turnover was low in all muscles, as described before for innervated conditions, with no change detected in *Mbnl1*^*ΔE3/ΔE3*^ muscles compared to controls (Fig. 4C). Similarly, there was no significant change in AChR turnover in TA and EDL muscles from *HSA*^*LR*^ mice (Fig. 4D). These results indicate that CaMKII deregulation, and more specifically the loss of CaMKIIβM, do not alter AChR dynamics in innervated DM1 muscle.

### Synaptic gene expression and muscle fibre type composition are altered in DM1 mouse models

**Fig. 5:**
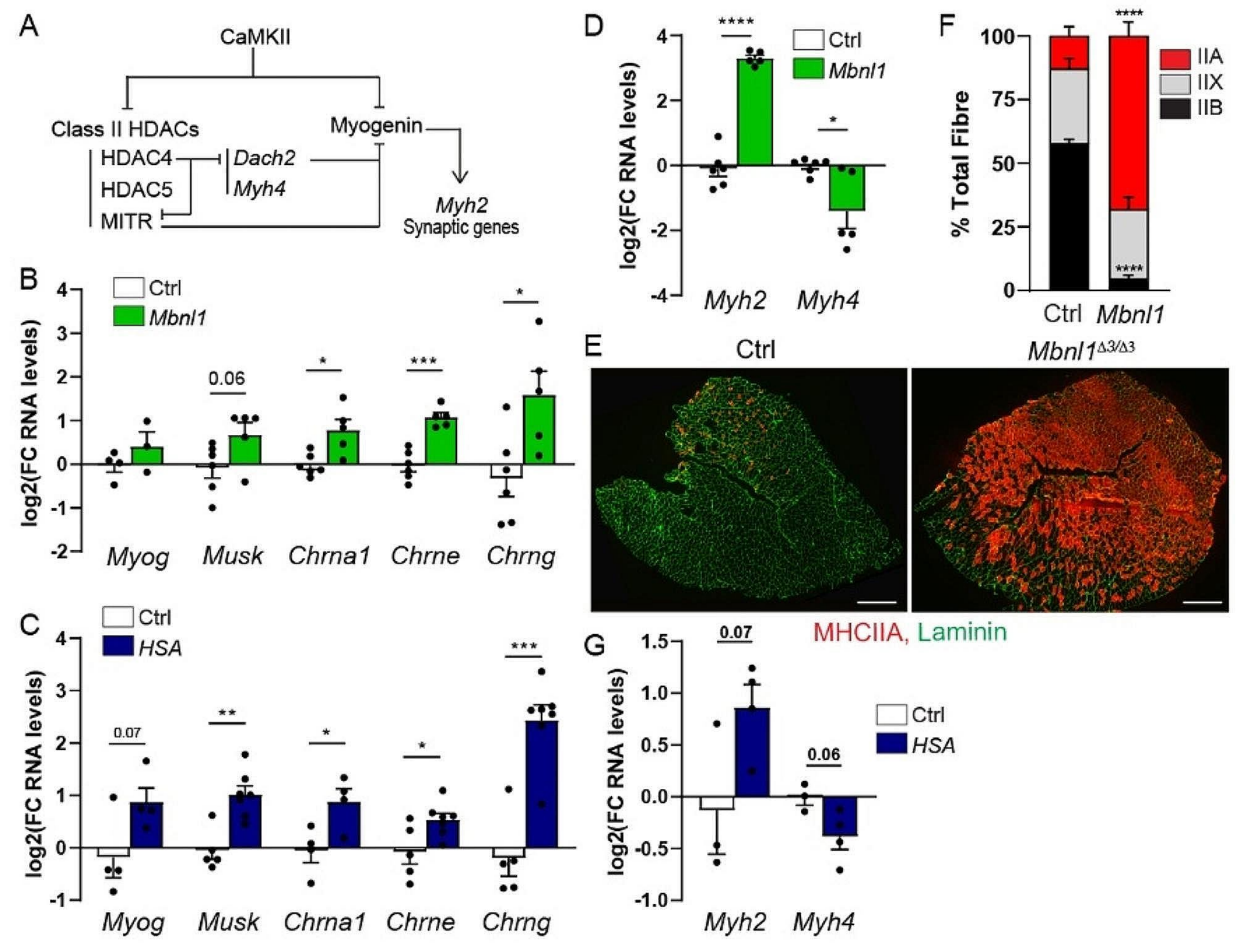
DM1 model mice display deregulation of activity-dependent signalling pathways. **A** Regulation of activity-dependent pathways by CaMKII. **B, C** Quantitative RT-PCR analysis of *Myog, Musk, Chrna1, Chrne* and *Chrng* in TA muscle from 3-month-old *Mbnl1*^*ΔE3/ΔE3*^ mice (B) and in *gastrocnemius* muscle from 3-month-old *HSA*^*LR*^ mice (C). *n* = 6/5 Ctrl/ *Mbnl1*^*ΔE3/ΔE3*^ (except for *Myog*, *n* = 4/3) and 5/7 Ctrl/*HSA*^*LR*^ (except for *Myog* and *Chrna1*, *n* = 4). **D** mRNA levels of *Myh2* and *Myh4*, encoding MHC2A and MHC2B, in *Mbnl1*^*ΔE3/ΔE3*^ TA muscle. *n* = 6/5 Ctrl/*Mbnl1*^*ΔE3/ΔE3*^. **E, F** Fluorescent images of control and *Mbnl1*^*ΔE3/ΔE3*^ muscles, stained with antibodies against MHC2A (red) and laminin (green), and quantification of the proportion of type IIA, IIX and IIB fibres in control and mutant muscles (F). Scale bar, 500 μm. **G** mRNA levels of *Myh2* and *Myh4* in *HSA*^*LR*^ TA muscle. *n* = 3/4 Ctrl/ *HSA*^*LR*^. All transcript levels (B, C, D, G) are normalized to *Tbp*, relative to control and expressed as log2(Fold Change). All data are mean ± SEM; * *p* < 0.05; ** *p* < 0.01; *** *p* < 0.001; ****p < 0.0001; two-tailed unpaired Student’s t-test

CaMKIIs are well known to mediate activity-dependent regulations (e.g., of synaptic gene expression) in adult skeletal muscle [34]. To assess the consequences of CaMKII deregulation in DM1 muscle, we first measured mRNA levels of *Myog*, which encodes myogenin, and of the synaptic genes *Musk*, *Chrna1*, *Chrne* and *Chrng* genes, which encode MUSK and the α, ε and γ subunits of AChR, respectively. During muscle development, expression of *Musk* and *Chrna1* becomes restricted to sub-synaptic myonuclei upon muscle innervation [35]. Simultaneously, *Chrng* transcripts are downregulated, while *Chrne* starts to be expressed in sub-synaptic nuclei (AChRε subunits replace AChRγ subunits). In non-synaptic regions of muscle fibres, synaptic gene repression is mediated by CaMKIIs and dependent on HDAC4/5 and myogenin inhibition (Fig. 5A) [8, 36]. This adult gene expression pattern depends on neural activity, as denervation reverts it back to a developmental pattern. Transcript levels of *Myog* tended to be higher in *Mbnl1*^*ΔE3/ΔE3*^ TA and *HSA*^*LR*^*gastrocnemius* innervated muscles, compared to control muscles (Fig. 5B, C). In parallel, transcript levels of *Musk, Chrna1, Chrne* and *Chrng* were strongly increased in both mutant muscles (Fig. 5B, C). Their levels were less or not changed in EDL and TA muscles from *HSA*^*LR*^ mice (Fig. S7A, B).

Expression of *Myh* genes, which encode myosin heavy chains (MHC), is also dependent on neural activity and Ca^2+^-associated signalling [37]. In particular, by inhibiting the activity of myogenin and HDAC4, CaMKIIs may perturb the expression of *Myh2* and *Myh4*, encoding MHCIIA and MHCIIB, respectively (Fig. 5A). Indeed, HDAC4 was shown to regulate the switch to type IIA fibres in TA muscle after nerve injury, by indirectly promoting the expression of *Myh2* and directly repressing *Myh4* [8]. Expression of *Myh2* was strongly increased in *Mbnl1*^*ΔE3/ΔE3*^ innervated muscle, as compared to control (Fig. 5D). In contrast, *Myh4* transcript levels were reduced in mutant muscle (Fig. 5D). Consistently, innervated TA muscle from *Mbnl1*^*ΔE3/ΔE3*^ mice displayed major accumulation of type IIA fibres and a loss of type IIB fibres compared to controls (Fig. 5E, F). Similarly, we previously described a mild switch towards type IIA fibres in TA muscle from *HSA*^*LR*^ mice [25]. There was also a tendency towards increased *Myh2* transcript levels and reduced expression of *Myh4* in *HSA*^*LR*^ TA muscle, as compared to controls (Fig. 5G). These results were in line with recent data describing myotonia-dependent switch towards oxidative muscle [38]. These results highlight that activity-dependent regulations underlying synaptic gene expression and fibre type composition, are perturbed in muscles from DM1 mouse models, which may involve CaMKII deregulation.

### HDAC4 accumulates in *Mbnl1*^*ΔE3/ΔE3*^ and *HSA*^*LR*^ muscles

**Fig. 6:**
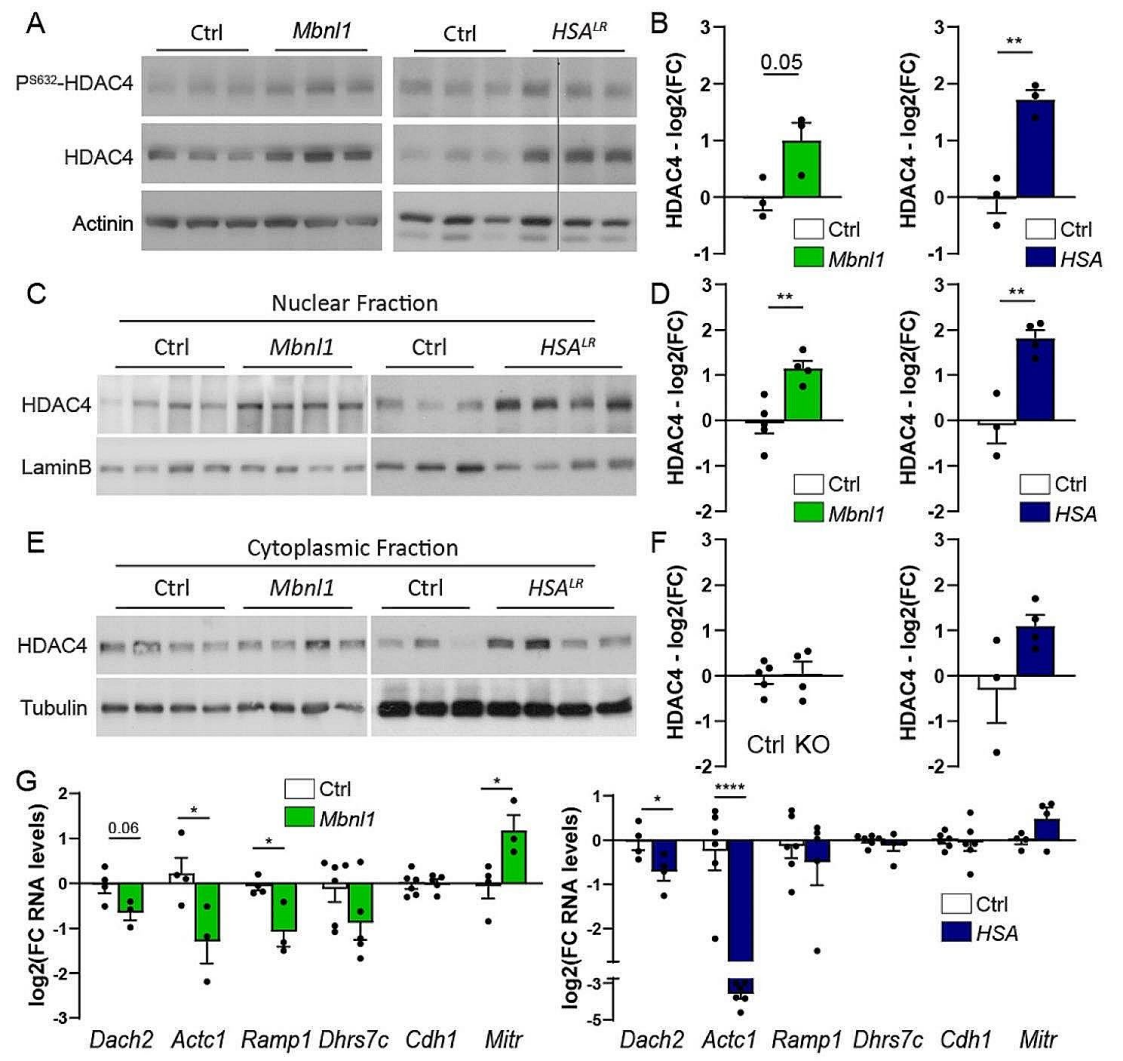
Changes in HDAC4 signalling pathway in *Mbnl1*^*ΔE3/ΔE3*^ and *HSA*^*LR*^ muscles. **A, B** Western blot analysis of HDAC4 and its phosphorylated form (Ser632) in total protein lysate of *Mbnl1*^*ΔE3/ΔE3*^ and *HSA*^*LR*^ muscles. Quantification of total levels is given in B. Quantification of phosphorylated levels is given in Supplementary Material, Fig. S8B. Total levels are normalized to α-actinin. *n* = 3 per group. **C, D** Western blot analysis of HDAC4 in nuclear (C, D) and cytosolic (E, F) fractions of *gastrocnemius* muscle from *Mbnl1*^*ΔE3/ΔE3*^ mice and *HSA*^*LR*^ mice. Quantifications of HDAC4 levels are given in D and F. Levels are normalized to lamin-B (D) and tubulin (F). *n* = 5/4 Ctrl/*Mbnl1*^*ΔE3/ΔE3*^ and 3/4 Ctrl/*HSA*^*LR*^. **G** mRNA levels of *Dach2, Actc1, Ramp1, Dhrs7c, Cdh1* and *Mitr* in TA muscle from *Mbnl1*^*ΔE3/ΔE3*^ mice and in *gastrocnemius* muscle from *HSA*^*LR*^ mice. Transcript levels are normalized to *Tbp*. *n* = 4/3 Ctrl/*Mbnl1*^*ΔE3/ΔE3*^ (except for *Dhrs7c* and *Cdh1*, *n* = 6/5) and 4/4 (*Dach2/Mitr*), 6/6 (*Actc1/Cdh1*), 6/5 (*Ramp1/Dhrs7c*) Ctrl/*HSA*^*LR*^. All protein (B, D, F) and RNA (G) levels are relative to control and expressed as log2(Fold Change). All data are mean ± SEM; * *p* < 0.05; ** *p* < 0.01; ****p < 0.0001; two-tailed unpaired Student’s t-test

CaMKIIs inhibit the nuclear import, and thereby the activity of HDACs, such as HDAC4 [39]. To determine whether changes in HDAC4 pathway mediate the effect of CaMKII deregulation on activity-dependent regulations in DM1 mouse models, we analysed the expression of HDAC4 and of its target genes in *Mbnl1*^*ΔE3/ΔE3*^ and *HSA*^*LR*^ muscles. While transcript levels of *Hdac4* were unchanged in *Mbnl1*^*ΔE3/ΔE3*^ and *HSA*^*LR*^ muscles (Fig. S8A), HDAC4 protein levels were higher in mutant muscles, as compared to controls (Fig. 6A, B). CaMKII-dependent phosphorylation of HDAC4 (Ser632) remained unchanged in *Mbnl1*^*ΔE3/ΔE3*^ muscle, but decreased in *HSA*^*LR*^ muscle (Fig. 6A and Fig. S8B). Notably, HDAC4 accumulated in nuclear fractions of *Mbnl1*^*ΔE3/ΔE3*^ and *HSA*^*LR*^ muscles (Fig. 6C, D). In contrast, HDAC4 protein levels were unchanged in cytosolic fractions of mutant muscles (Fig. 6E, F). This suggests that HDAC4 nuclear import is increased in *Mbnl1*^*ΔE3/ΔE3*^ and *HSA*^*LR*^ muscles. HDAC4 remained, however, barely detectable in mutant muscles by immunostaining (Fig. S8C). To assess whether HDAC4 accumulation translates into higher nuclear activity, we next evaluated the expression of target genes directly repressed by HDAC4. We focused on *Dach2* and *Mitr* [36, 40], as well as on *Ramp2 (Receptor Activity Modifying Protein 2), Actc1 (Actin alpha cardiac muscle 1), Cdh1 (Cadherin 1)* and *Dhrs7c* (*Dehydrogenase/Reductase 7 C)*, which were identified in public RNA-seq data as HDAC4 targets repressed after denervation [41]. Consistent with higher HDAC4 activity, transcript levels of *Dach2, Actc1, Ramp1*, and to a lesser extent of *Dhrs7c*, were reduced in *Mbnl1*^*ΔE3/ΔE3*^ and/or *HSA*^*LR*^ muscles (Fig. 6G), as observed for *Myh4* (Fig. 5D, G). However, the expression of other genes was unchanged (*Cdh1*) or increased (*Mitr*) in mutant muscles compared to controls (Fig. 6G), suggesting incomplete or mild increase in HDAC4 activity. Hence, nuclear HDAC4 accumulation in skeletal muscle from DM1 mouse models may contribute to transcriptional changes of some activity-dependent genes downstream of CaMKII deregulation in innervated conditions.

### *Mbnl1*^*ΔE3/ΔE3*^ mice show resistance to denervation-induced muscle atrophy

Perturbations observed in innervated muscle from DM1 mouse models are reminiscent of the changes induced after denervation (i.e., synaptic gene up-regulation, HDAC4 accumulation, fibre type switch). CaMKII deregulation in mutant mice may interfere with the effect of neural activity in skeletal muscle. In turn, it may impair the muscle response to neural inactivity and reduce adaptive changes in activity-dependent processes. This reduced muscle plasticity may contribute to progressive NMJ deterioration observed in DM1 mouse models. To test this hypothesis, we challenged activity-dependent signalling pathways in DM1 mouse models with nerve injury. To this end, we cut the sciatic nerve of 3-month-old *Mbnl1*^*ΔE3/ΔE3*^ and *HSA*^*LR*^ mice to obtain complete denervation of hind limb muscles. Unexpectedly, we observed that *HSA*^*LR*^ mice lose the expression of the *HSA* transgene after 3 days of denervation (Fig. S9A). Consequently, ribonuclear foci accumulation and *Clcn1* mis-splicing were reduced in denervated *HSA*^*LR*^ muscle, as compared to innervated muscle (Fig. S9B, C). Therefore, we limited the analysis to the *Mbnl1*^*ΔE3/ΔE3*^ mouse line.

**Fig. 7:**
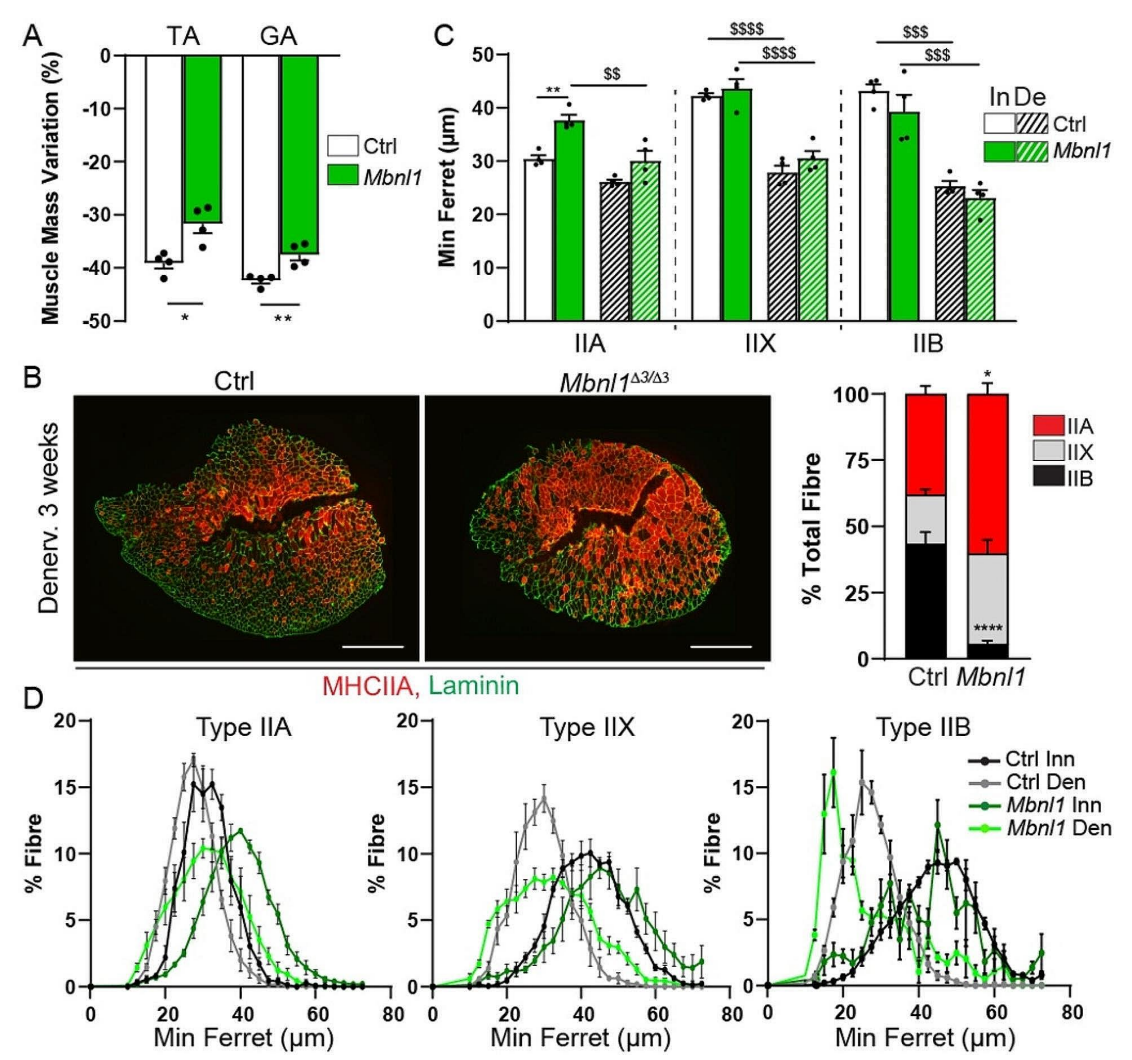
*Mbnl1*^*ΔE3/ΔE3*^ muscle shows resistance to atrophy upon denervation. **A** Mass variation after 3 weeks of denervation in control and *Mbnl1*^*ΔE3/ΔE3*^ mice, for TA and *gastrocnemius* (GA) muscles. *n* = 4 per group. **B** Fluorescent images of denervated muscles from control and *Mbnl1*^*ΔE3/ΔE3*^ mice, stained with antibodies against type IIA myosin heavy chain (MHC2A - red) and laminin (green) and quantification of fibre type proportion in control and mutant denervated muscles. Scale bar, 500 μm. *n* = 4 mice per group. **C** Quantification of fibre minimum ferret in innervated and 3-week-denervated muscles from control and *Mbnl1*^*ΔE3/ΔE3*^ mice. *n* = 4 mice per group. **D** Fibre size distribution for type IIA, IIX and IIB fibres in innervated and 3-week-denervated muscles from *Mbnl1*^*ΔE3/ΔE3*^ and control mice. *n* = 4/4 Ctrl/*Mbnl1*^*ΔE3/ΔE3*^. All data are mean ± SEM; ^$^*p* < 0.05, ^$$^*p* < 0.01, ^$$$^*p* < 0.001, ^$$$$^*p* < 0.0001 between Inn/Den; * *p* < 0.05, ** *p* < 0.01, **** *p* < 0.0001 between genotypes; two-way ANOVA with a Tukey’s post-hoc analysis (Inn/Den and Ctrl/ *Mbnl1*^*ΔE3/ΔE3*^; C) or two-tailed unpaired Student’s t-test (A, B)

After denervation, the loss of muscle mass was significantly less in *Mbnl1*^*ΔE3/ΔE3*^ mice, as compared to control mice (Fig. 7A). Nerve injury did not aggravate muscle degeneration in *Mbnl1*^*ΔE3/ΔE3*^ mice (Fig. S10A). To get insights into the mechanisms of this atrophy resistance, we first evaluated changes in pathways known to contribute to muscle atrophy after denervation. Transcript levels of the atrogenes *Fbxo32* and *Trim63*, which are induced by HDAC4 and FoxO pathways after nerve injury [42, 43], were similar in *Mbnl1*^*ΔE3/ΔE3*^ and control muscles 3 days after denervation (Fig. S10B). We and others have reported that the anabolic pathway Akt/mTORC1 (*mammalian Target Of Rapamycin Complex 1*) is deregulated in DM1 muscle [25], and that its activation contributes to muscle atrophy upon denervation [23, 43]. Thus, we assessed whether Akt/mTORC1 activity is perturbed in DM1 denervated muscle. At 3 days of denervation, there was no change in the levels of the phosphorylated, active form of Akt (Akt^P473^) in *Mbnl1*^*ΔE3/ΔE3*^ and control muscles, and levels of the active form of S6 (S6^P235^) increased similarly in both denervated muscles (Fig. S10C). Moreover, levels of the autophagic marker LC3II remained largely unchanged in *Mbnl1*^*ΔE3/ΔE3*^ and control muscles, suggesting that autophagy is not strongly affected (Fig. S10C). These results suggest that atrophy resistance does not arise from perturbations in atrogene expression or autophagy after denervation in DM1 muscle. We next evaluated muscle fibre type and size after 3 weeks of denervation. Control TA muscle shifted to type IIA fibres upon denervation, approaching the fibre type composition observed in *Mbnl1*^*ΔE3/ΔE3*^ innervated muscle (Fig. 7B). Fibre type proportion remained unchanged in *Mbnl1*^*ΔE3/ΔE3*^ denervated muscle (Fig. 7B). The minimum ferret of type IIA fibres was increased in *Mbnl1*^*ΔE3/ΔE3*^ innervated muscle as compared to control innervated muscle, but it decreased upon denervation in mutant mice (Fig. 7C, D). In parallel, the size of type IIX and IIB fibres was similar in *Mbnl1*^*ΔE3/ΔE3*^ and control innervated muscles, and strongly decreased upon denervation in both mutant and control mice (Fig. 7C, D). Hence, the predominance of type IIA fibres in mutant mice and their relative resistance to denervation-induced atrophy as compared to type IIX/B fibres (Fig. 7D) may explain why *Mbnl1*^*ΔE3/ΔE3*^ mice show limited muscle mass loss upon nerve injury.

### Endplate remodelling is impaired in *Mbnl1*^*ΔE3/ΔE3*^ muscle upon denervation

**Fig. 8:**
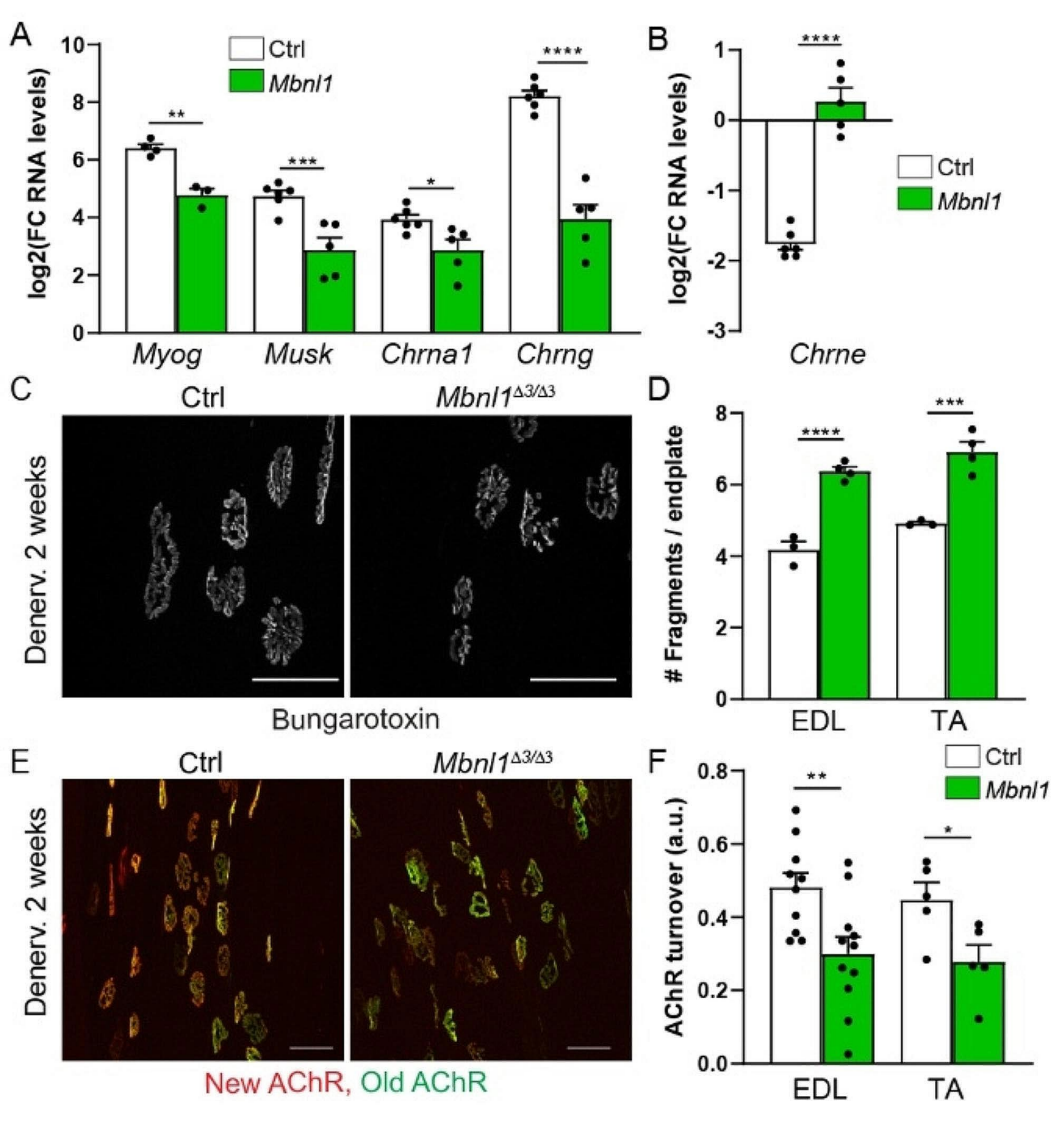
Denervation-induced changes in the expression and dynamics of synaptic proteins are impaired in *Mbnl1*^*ΔE3/ΔE3*^ mice. **A, B** mRNA levels of *Myog, Musk, Chrna1* and *Chrng* (A) and *Chrne* (B) in TA muscle from control and *Mbnl1*^*ΔE3/ΔE3*^ mice 3 days post-denervation. Levels are normalized to *Tbp*, relative to control innervated muscle and expressed as log2(Fold Change). *n* = 6/5 Ctrl/ *Mbnl1*^*ΔE3/ΔE3*^ (except for *Myog*, *n* = 4/3). **C, D** Fluorescent images of endplates stained with α-bungarotoxin in EDL muscle from control and *Mbnl1*^*ΔE3/ΔE3*^ mice 2 weeks post-denervation. Quantification of endplate fragmentation in EDL and TA muscles is given in D. *n* = 3/4 Ctrl/KO. Scale bar, 50 μm. **E, F** AChR turnover in denervated muscle from *Mbnl1*^*ΔE3/ΔE3*^ and control mice. Fluorescent images of “old” and “new” receptors are shown in (E). Scale bar, 50 μm. Quantification of AChR turnover is given in (F). *n* = 10/11 (EDL) and 5/5 (TA) Ctrl/*Mbnl1*^*ΔE3/ΔE3*^. Data are mean ± SEM; *p < 0.05; ** *p* < 0.01; *** *p* < 0.001; ****p < 0.0001 between genotypes; two-way ANOVA with a Tukey’s post-hoc analysis (Inn/Den and Ctrl/ *Mbnl1*^*ΔE3/ΔE3*^)

Synaptic remodelling after denervation includes a strong increase in AChR turnover in the sub-synaptic region, together with synaptic gene up-regulation throughout the fibre [44]. The release of synaptic gene repression in non-synaptic muscle regions depends on HDAC4 induction, which may rely on CaMKII inhibition [8, 36]. To further characterize the muscle response to denervation in *Mbnl1*^*ΔE3/ΔE3*^ mice, we first evaluated the expression of synaptic genes 3 days after nerve injury. Importantly, the up-regulation of *Myog*, *Musk, Chrna1* and *Chrng* was hampered in mutant TA muscle, as compared to controls (Fig. 8A). Inversely, the expression of *Chrne* remained abnormally high in *Mbnl1*^*ΔE3/ΔE3*^ muscle upon denervation (Fig. 8B). This defect occurred despite an efficient induction of HDAC4 in mutant mice. Indeed, there was a strong increase in the transcript (Fig. S11A) and protein (Fig. S11B) levels of HDAC4 in denervated muscle from both mutant and control mice. Consistently, HDAC4 accumulated in myonuclei of *Mbnl1*^*ΔE3/ΔE3*^ and control denervated muscles (Fig. S11C). Of note, changes in CaMKII expression pattern persisted upon denervation in *Mbnl1*^*ΔE3/ΔE3*^ muscle (Fig. S11B). Expression of the direct targets of HDAC4, *Dach2* and *Mitr*, was similarly repressed in *Mbnl1*^*ΔE3/ΔE3*^ and control denervated muscles (Fig. S11D).

To assess the consequences of the defective up-regulation of synaptic genes in mutant muscle, we evaluated changes at the endplate after 2 weeks of denervation. At this stage, endplate fragmentation increased and remained higher in EDL and TA muscles from *Mbnl1*^*ΔE3/ΔE3*^ mice, compared to controls *(*Fig. 8C, D). We next quantified AChR turnover by labelling receptors 5 days after nerve injury and assessing their turnover 10 days later. In control muscle, AChR turnover increased drastically after denervation (Fig. 8E, F), as previously reported [45]. Interestingly, AChR turnover was reduced in TA and EDL denervated muscles from *Mbnl1*^*ΔE3/ΔE3*^ mice, as compared to control denervated muscle (Fig. 8E, F). Reduced AChR turnover in mutant muscle may arise from the defective expression of synaptic genes and reduced insertion of AChRs. Together, these results point to an impaired response to denervation and reduced plasticity to neural (in)activity of *Mbnl1*^*ΔE3/ΔE3*^ muscle.

### CaMKIIβ/βM overexpression normalizes endplate fragmentation and synaptic gene expression in innervated *Mbnl1*^*ΔE3/ΔE3*^ muscle

**Fig. 9:**
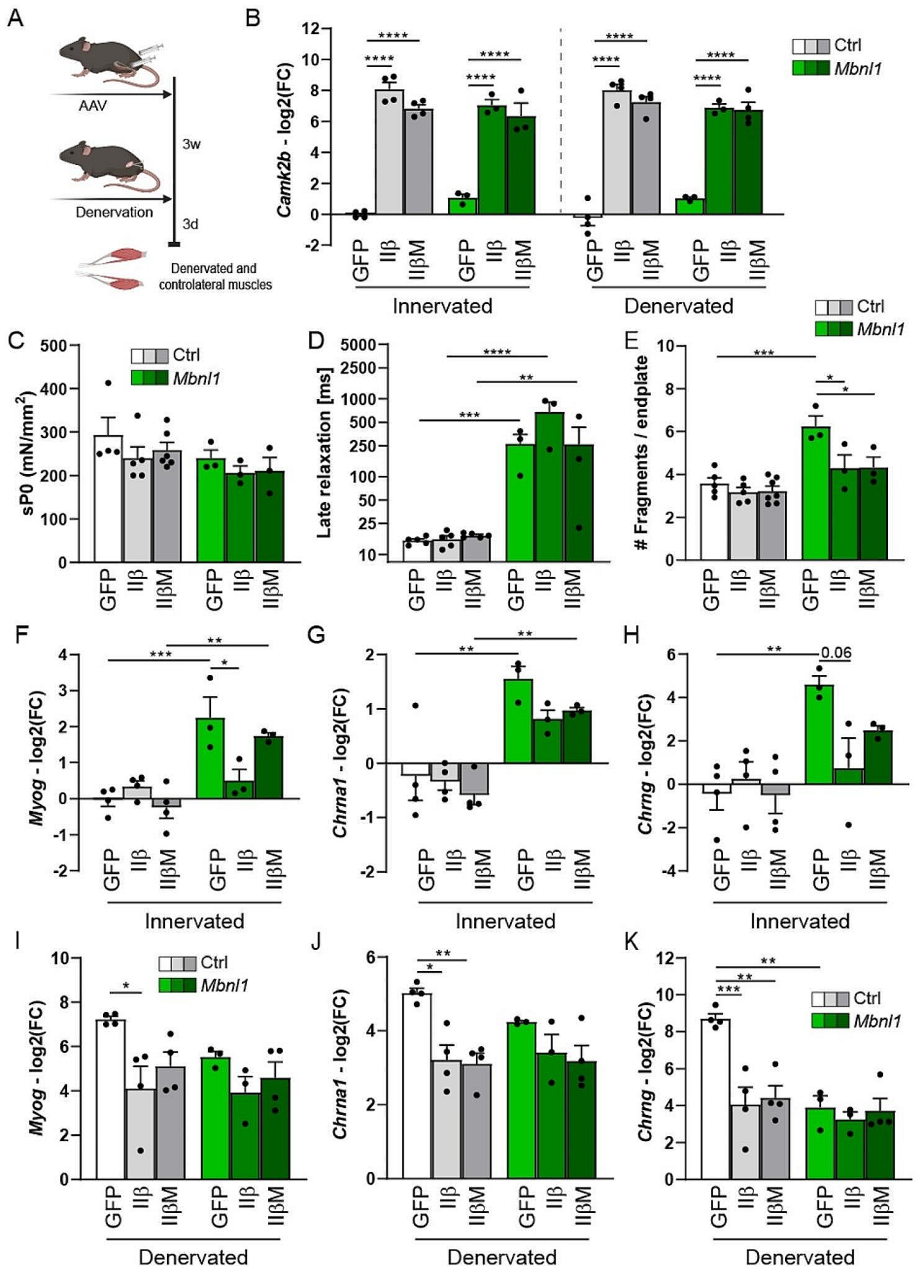
CaMKIIβ/βM overexpression normalizes endplate fragmentation in *Mbnl1*^*ΔE3/ΔE3*^ innervated muscle. **A** AAV-based overexpression strategy to evaluate the role of CaMKIIβ/βM deregulation in *Mbnl1*^*ΔE3/ΔE*^ mice. Created with Biorender.com. **B** mRNA levels of *Camk2b* in control and *Mbnl1*^*ΔE3/ΔE3*^ TA muscles infected with AAV-GFP, -CaMKIIβ, or -CaMKIIβM. Levels are normalized to *Tbp*, relative to control innervated muscle and expressed as log2(Fold Change). *n* = 4 Ctrl and 3 *Mbnl1*^*ΔE3/ΔE3*^ (except in Den, *n* = 4 IIβM). **C, D** Specific tetanic force (sP0; C) and late relaxation time (D) upon stimulation of control and *Mbnl1*^*ΔE3/ΔE3*^ EDL muscles infected with AAV-GFP, -CaMKIIβ, or -CaMKIIβM. *n* = 4/5/6 Ctrl and 3 *Mbnl1*^*ΔE3/ΔE3*^ per group. **E** Number of fragments per endplate in control and *Mbnl1*^*ΔE3/ΔE3*^ EDL innervated muscles injected with AAV-GFP, -CaMKIIβ, or -CaMKIIβM. *n* = 5/5/7 Ctrl and 3 *Mbnl1*^*ΔE3/ΔE3*^ per group. **F-K** mRNA levels of *Myog* (F, I), *Chrna1* (G, J) and *Chrng* (H, K) in control and *Mbnl1*^*ΔE3/ΔE3*^ TA innervated (F-H) and denervated (3 days; I-K) muscles injected with AAV-GFP, -CaMKIIβ, or -CaMKIIβM. Levels are normalized to *Tbp*, relative to control innervated muscle and expressed as log2(Fold Change). *n* = 4 Ctrl and 3 *Mbnl1*^*ΔE3/ΔE3*^ (except for Den IIβM, *n* = 4). Data are mean ± SEM; *p < 0.05; ** *p* < 0.01; *** *p* < 0.001; ****p < 0.0001; two-way ANOVA with Tukey’s post-hoc correction

To determine the contribution of CaMKII deregulation in endplate fragmentation and changes in synaptic gene expression observed in *Mbnl1*^*ΔE3/ΔE3*^ mice, we assessed the consequences of CaMKII modulation in mutant muscle. As DM1 mouse models shifted from CaMKIIβ/βM to CaMKIIβe expression, we injected isotype 9 adeno-associated virus (AAV9) in the anterior compartment of control and *Mbnl1*^*ΔE3/ΔE3*^ mice to overexpress either CaMKIIβ or CaMKIIβM. Three weeks later, we unilaterally denervated the mice and harvested innervated and denervated muscles 3 days later (Fig. 9A). A single AAV injection was sufficient to strongly increase the expression of CaMKIIβ/βM in TA innervated and denervated muscles, as compared to muscle injected with AAV-GFP (Fig. 9B). CaMKIIβ/βM overexpression did not modify TA muscle mass normalized to body weight, as compared to GFP-overexpressing muscle (Fig. S12A). Moreover, it did not alter muscle histology in control and mutant mice (Fig. S12B). In parallel, the specific tetanic force (Fig. 9C) and the late relaxation time (Fig. 9D) of EDL innervated control and mutant muscles were unchanged upon CaMKIIβ/βM overexpression, as compared to GFP-overexpressing muscle.

Importantly, overexpression of CaMKIIβ or CaMKIIβM was sufficient to normalize endplate fragmentation in *Mbnl1*^*ΔE3/ΔE3*^ EDL innervated muscle, as compared to control (Fig. 9E and Fig. S12C). CaMKIIβ/βM overexpression did not perturb synaptic gene expression in control innervated muscle (Fig. 9F-H). Conversely, CaMKIIβ overexpression normalized *Myog* expression in mutant innervated muscle (Fig. 9F) and tended to reduce *Chrna1* and *Chrng* expression (Fig. 9G, H), as compared to GFP-overexpressing mutant muscle. CaMKIIβM overexpression showed the same trend but was less efficient (Fig. 9F-H). In control muscle, CaMKIIβ/βM overexpression was sufficient to reduce the up-regulation of *Myog*, *Chrna1* and *Chrng* upon denervation (Fig. 9I-K). In contrast, it did not further reduce synaptic gene expression in *Mbnl1*^*ΔE3/ΔE3*^ denervated muscle (Fig. 9I-K). Together, these results indicate that CaMKIIβ/βM deficiency contributes to synaptic gene deregulation and endplate fragmentation in innervated *Mbnl1*^*ΔE3/ΔE3*^ muscles, but not to their defective response to denervation. To determine whether the effect of CaMKIIβ/βM overexpression involves changes in HDAC4 pathway, we evaluated the expression of HDAC4 target genes. CaMKIIβ/βM overexpression normalized the expression of *Dach2* and *Ramp1* in *Mbnl1*^*ΔE3/ΔE3*^ innervated muscle, as compared to control muscle (Fig. S13A). Moreover, it restricted the repression of *Dach2* and *Mitr* after denervation in control mice (Fig. S13A). Strikingly, denervation-induced HDAC4 accumulation was abrogated by CaMKIIβ/βM overexpression in control and mutant mice (Fig. S13B), which was consistent with the defective repression of HDAC4 target genes. These changes suggest that HDAC4 inhibition mediates some of the transcriptional changes observed in control and mutant mice after CaMKIIβ/βM overexpression.

## Discussion

Although mis-splicing events are at the basis of DM1 pathogenesis, how the consecutive perturbations lead to muscle alterations is unclear. NMJ deteriorations have been described in muscle biopsies from DM1 patients and in muscle from DM1 mouse models [10–14, 46]. However, the mechanisms underlying these perturbations and whether these defects arise from DM1-related changes in the muscle or in the nerve remain unknown. Here, we showed that NMJs are affected in *Mbnl1*^*ΔE3/ΔE3*^ and *HSA*^*LR*^ mice, two well-characterized mouse models of DM1. We established that CaMKIIs and activity-dependent signalling pathways are disrupted in *Mbnl1*^*ΔE3/ΔE3*^ and *HSA*^*LR*^ muscles, which may contribute to endplate destabilization and to the abnormal response of mutant muscles to denervation.

Signs of NMJ deterioration, without denervation of muscle fibres, have been reported in muscle biopsies from DM1 patients, as well as in DMSXL and *Mbnl1/2*-deficient mice [10–14, 46]. As motor neurons are also affected in these mouse models, it is unclear whether the defects arise from pre- or post-synaptic perturbations. Here, we found endplate fragmentation in the muscles of *Mbnl1*^*ΔE3/ΔE3*^ and *HSA*^*LR*^ mice at different ages. As *HSA*^*LR*^ mice express the transgene carrying the CTG repeats only in muscle, perturbations in the post-synaptic compartment, i.e. the muscle, are likely responsible for NMJ deterioration in DM1. Importantly, endplate fragmentation was similar between TA, EDL and *gastrocnemius* muscles, which are affected differentially in *HSA*^*LR*^ mice. In particular, TA muscle showed only few signs of muscle degeneration, suggesting that endplate destabilization is a primary defect in DM1. NMJ deterioration may in fact contribute to and precede muscle atrophy, weakness and fatigue observed in DM1 muscle.

To determine the pathomechanisms that compromise NMJ integrity in DM1, we examined the potential role of CaMKIIs. Mis-splicing in *Camk2b, 2d* and *2 g* has been reported in DM1 patients, as well as in mouse models [3–5]. In particular, exclusion of *Camk2b* exon 13 appeared as one of the most important splicing changes detected in DM1 tissues. Although the pathophysiological consequences have been investigated in the brain, the consequences of CaMKII deregulation in skeletal muscle have not yet been analysed. Here, we report that, together with exon 13 exclusion, the three exons specifically included in the muscle-specific isoform of CaMKIIβ are excluded in *HSA*^*LR*^ and *Mbnl1*^*ΔE3/ΔE3*^ muscles. This indicates that *Camk2b* splicing is MBNL1-dependent. Of note, in *HSA*^*LR*^ mice, *Camk2b* mis-splicing was detected in *gastrocnemius*, TA and EDL muscles. Together with exons 13 and 16, exons 18–20 are part of the highly variable region of the *Camk2b* gene, which allows the expression of tissue-specific variants. Especially, CaMKIIβM was shown to be the only isoform accumulating at the NMJ [6]. Consistent with the abnormal splicing of *Camk2b* in DM1 muscle, CaMKIIβM was not detected in *Mbnl1*^*ΔE3/ΔE3*^ and HSA^LR^ muscles. CaMKIIs have been shown to contribute to synaptic gene repression in non-synaptic regions of muscle fibres, by inhibiting myogenin activity and HDAC4 signalling pathway [7, 36]. We reveal that HDAC4 accumulates in myonuclei from *Mbnl1*^*ΔE3/ΔE3*^ and *HSA*^*LR*^ muscles, which was not caused by spontaneous denervation of mutant muscles. CaMKII deficiency and the consecutive increase in HDAC4 activity may hence contribute to the up-regulation of synaptic genes and to the fibre type switch observed in mutant muscles. As some HDAC4 targets remained unchanged in mutant muscles, changes in synaptic genes detected in DM1 muscle may occur predominantly at the endplate, as their regulation there is independent from HDAC4/myogenin in innervated muscle [35]. Alternatively, abnormal accumulation of other CaMKII isoforms, such as CaMKIIβe, may inhibit the repressor MITR/HDAC9 and thereby mediate some of the effects observed on activity-dependent genes. Importantly, we show that overexpression of CaMKIIβ, and to a lesser extent of CaMKIIβM, normalizes synaptic gene expression in innervated mutant muscle, consistent with a primary role of CaMKII deficiency in activity-dependent gene deregulation. Simultaneously, CaMKIIβ/βM overexpression reverses endplate fragmentation in mutant muscle. As synaptic gene up-regulation was detected in *gastrocnemius* muscle, but not in TA muscle from *HSA*^*LR*^ mice, it is unlikely that it underlies endplate fragmentation that was observed in all muscles. Despite the known role of CaMKIIs in regulating AChR recycling, we did not find perturbation in AChR dynamics at the endplate in mutant muscle. CaMKII deregulation may alternatively increase endplate fragmentation by perturbing AChR clustering at the endplate, as suggested by previous studies [6, 47]. Of note, the fibre type switch previously reported in *HSA*^*LR*^ muscle [25] was exacerbated in *Mbnl1*^*ΔE3/ΔE3*^ muscle. This suggested that perturbations both in the muscle and in non-muscle tissues (e.g., the motor neuron) contribute to these changes.

To obtain further insights into the capacity of DM1 muscle to regulate activity-dependent signalling and to maintain endplates, we challenged *Mbnl1*^*ΔE3/ΔE3*^ and *HSA*^*LR*^ mice with nerve injury. As transgene expression driven by the *HSA* promoter was lost in *HSA*^*LR*^ muscle upon denervation, we needed to limit the analysis to *Mbnl1*^*ΔE3/ΔE3*^ mice. In this model, denervation-induced increase in AChR turnover and synaptic gene up-regulation were hampered, suggesting that incorporation of new receptors at the endplate is limited in denervated mutant muscle. Synaptic gene induction upon denervation relies on the release of *Myog* expression, through the repression of *Mitr/Hdac9* and *Dach2* by HDAC4 [8, 48, 49]. Expression of these two repressors was efficiently reduced upon denervation in DM1 muscle, which was consistent with the major accumulation of HDAC4 in mutant denervated muscle. In addition, CaMKIIβ/βM overexpression did not restore synaptic gene expression in denervated mutant muscles, but rather decreased synaptic gene up-regulation in control denervated muscle. Hence, the incomplete induction of synaptic genes in *Mbnl1*^*ΔE3/ΔE3*^ denervated muscle is unlikely to occur *via* HDAC4 signalling deregulation or CaMKIIβ/βM deficiency. However, we cannot rule out contribution of other CaMKII isoforms to the defective muscle response to denervation of *Mbnl1*^*ΔE3/ΔE3*^ mice. Changes in the activity of other repressors of *Myog*, such as Msy3/Ybx3 [50] or CtBP1 [51], may also be involved in the defects observed. Finally, ClC-1 deregulation may hinder the response to denervation in DM1 muscle, as primary changes triggered by nerve injury at the endplate have been associated with chloride influx [52]. Consistently, another myotonic mouse model deficient for ClC-1 also displayed impaired muscle response to denervation [53].

## Conclusions

In conclusion, our study identified NMJ deterioration as an integral part of muscle dysfunction in DM1, which likely involves muscle perturbations in activity-dependent pathways. Especially, deregulation of CaMKII isoforms may be a key event in endplate destabilization, as well as in DM1 pathogenesis in muscle and non-muscle tissues.

### Electronic supplementary material

Below is the link to the electronic supplementary material.

Supplementary Material 1

## Data Availability

Data generated during the study are freely accessible on the Yareta repository database: 10.26037/yareta:qx7k7d2vcva2fo7jr5t3bn2bly. RNAseq data are available under the GEO accession number: GSE226676.
