# Supplementary material for: CaMKIIβ deregulation contributes to neuromuscular junction destabilization in Myotonic Dystrophy type I
Source: Skelet Muscle. 2024 May 21;14:11. doi: 10.1186/s13395-024-00345-3 (PMC11106974; doi:10.1186/s13395-024-00345-3)
Supplement: Supplementary file 1 — Supplementary Material 1 [file 13395_2024_345_MOESM1_ESM.pdf]

Supplementary Data

CaMKIIβ deregulation contributes to neuromuscular junction destabilization in Myotonic Dystrophy type I

Denis Falcetta, Sandrine Quirim, Ilaria Cocchiararo, Florent Chabry, Marine Théodore, Adeline Stiefvater, Shuo Lin, Lionel Tintignac, Robert Ivanek, Jochen Kinter, Markus A. Rüegg, Michael Sinnreich, Perrine Castets

Supplementary data includes: Supplementary Material, 13 Supplementary Figures, 1 Supplementary Table.

SUPPLEMENTARY MATERIAL

Antibodies

The following antibodies were used for immunoblotting (dilution 1/1000) or immunoflurescence: HDAC4 (#15164 and #7628; 1/5000 for IHC), Phospho-HDAC4Ser632 (#3424), Phospho-CaMKIIThr286/287 (#12716), CaMKII pan (#4436), Akt (#9272), Phospho-Akt^{Ser473} (#9271), S6 Ribosomal Protein (#2217), Phospho-S6 Ribosomal Protein$^{Ser235/6}$ (#2211), LC3B (#2775), GAPDH (#2118) from Cell Signaling Technology; α-actinin (A5044), Acetylcholinesterase (SAB4200839) and Neurofilament 200 (N4142; 1/2000 for IHC) from Sigma; Laminin (ab11575 and ab11576; 1/300 for IHC) and Tubulin (ab15246) from Abcam; Synaptophysin (A0010; 1/200 for IHC) from Dako; myosin heavy chain IIA (A4.74) and IIB (BF.F3) from Developmental Studies Hybridoma Bank; Lamin-B (sc-6216) from Santa Cruz Biotechnology.

Fluorescence *in situ* hybridization

FISH was conducted on muscle cryosections as previously described by Batra et al. [1], using a Cy3-CAG10 DNA probe. Nuclear foci were observed with a Leica confocal microscope.

RNA-seq

Single-end RNA-seq reads were mapped to the mouse genome assembly (version mm10) with RNA-STAR (version 2.5.2a)[2], with default parameters, except for allowing up 10 hits to genome (outFilterMultimapNmax 10), for reporting only one location for hits with equal score (outSAMmultNmax 1), and for filtering reads without evidence in spliced junction table (outFilterType "BySJout"). All subsequent gene expression data analyses were done within the R software (R Foundation for Statistical Computing, Vienna, Austria). Raw reads and mapping quality were assessed by the qQCreport function from the R/Bioconductor software package QuasR (version 1.16.0)[3]. Using RefSeq mRNA coordinates from UCSC (*genome.ucsc.edu*, downloaded in December 2015) and the qCount function from QuasR package, we quantified gene expression as the number of reads that started within any annotated exon of a gene. The differentially expressed genes were identified using the edgeR package (version 3.18.1)[4]. The same RefSeq annotation was used to build the set of all existing junctions in transcripts, and qCount function was used to count the number of spanning reads in individual samples. The junctions were further filtered: only junction longer than 5 bp, and starting and ending in the same gene were kept, and those supported by at least 5 reads in total. The differentially used junctions were identified using the diffSpliceDGE function implemented in the edgeR package.

References

S1. Batra R, Charizanis K, Manchanda M, Mohan A, Li M, Finn DJ et al. Loss of MBNL leads to disruption of developmentally regulated alternative polyadenylation in RNA-mediated disease. Mol Cell. 2014; 56(2):311-322.

S2. Dobin A, Davis CA, Schlesinger F, Drenkow J, Zaleski C, Jha S et al. STAR: ultrafast universal RNA-seq aligner. Bioinformatics. 2013; 29(1):15-21.

S3. Gaidatzis D, Lerch A, Hahne F, Stadler MB. QuasR: quantification and annotation of short reads in R. Bioinformatics. 2015; 31(7):1130-1132.

S4. Robinson MD, McCarthy DJ, Smyth GK. edgeR: a Bioconductor package for differential expression analysis of digital gene expression data. Bioinformatics. 2010; 26(1):139-140.

S5. Wang ET, Treacy D, Eichinger K, Struck A, Estabrook J, Olafson H et al. Transcriptome alterations in myotonic dystrophy skeletal muscle and heart. Hum Mol Genet. 2019; 28(8):1312-1321.

Figure S1

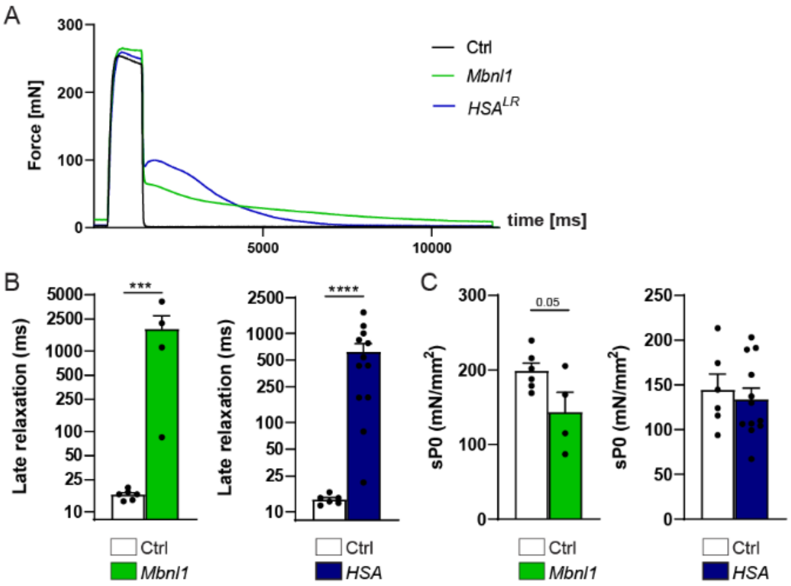

Figure S1, related to Figure 1: Myotonia and muscle force in DM1 mouse models. A, B Late relaxation time upon stimulation is increased in EDL muscle from *Mbnl1*$^{\Delta E3/\Delta E3}$ and *HSA*^{LR} mice, as compared to control mice. Quantification is given in B for 9-month-old *Mbnl1*$^{\Delta E3/\Delta E3}$ and 12-month-old *HSA*^{LR} mice. n = 6 Ctrl /4 *Mbnl1*$^{\Delta E3/\Delta E3}$; 6 Ctrl / 12 *HSA*^{LR}. **C** Specific tetanic force (sP0) of EDL muscle from 9-month-old *Mbnl1*$^{\Delta E3/\Delta E3}$ and 12-month-old *HSA*^{LR} mice. n = 6 Ctrl /4 *Mbnl1*$^{\Delta E3/\Delta E3}$; 6 Ctrl / 12 *HSA*^{LR}. All data are mean ± SEM; *** p<0.001; **** p<0.0001; two-tailed unpaired Student's t-test.

Figure S2

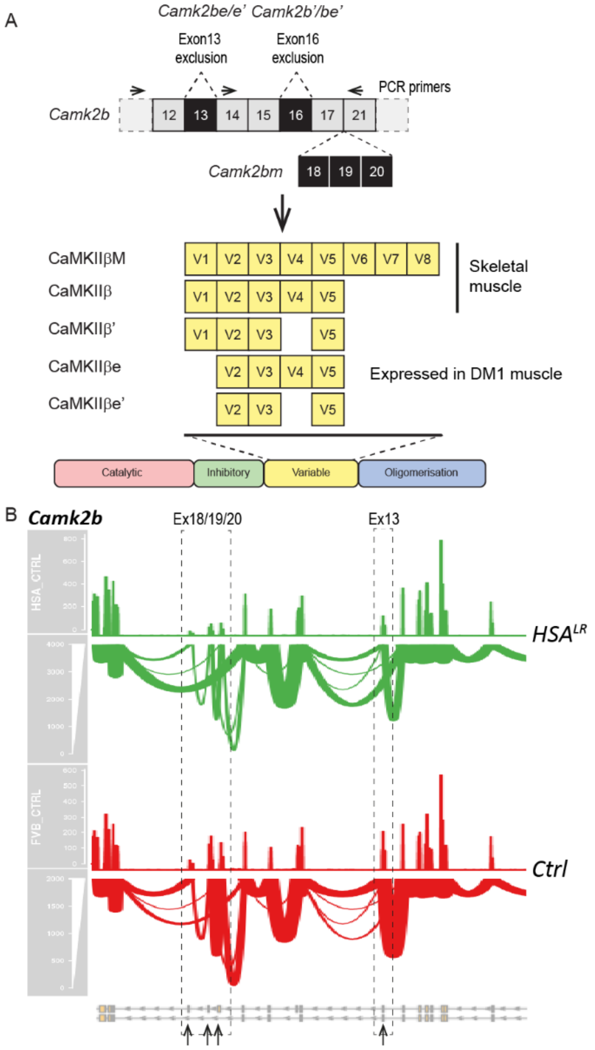

C

Camk2b					
Junctions	Start	End	log2FC	P.Value	adj.P.Val
Exon 12-14	5982762	5988100	1.800360763	2.609480e-48	1.748016e-44
Exon 17-21	5972889	5979672	1.205322257	8.284714e-38	3.641986e-34
Exon 17-18	5978513	5979672	-1.018415264	1.165818e-21	2.309846e-18
Exon 20-21	5972889	5976764	-1.013880194	9.656338e-21	1.741521e-17
Exon 13-14	5982759	5987056	-0.768667746	2.036010e-20	3.535947e-17
Exon 18-19	5977941	5978398	-1.066848895	4.964948e-18	6.589002e-15
Exon 12-13	5987132	5988100	-0.746184903	4.399391e-17	5.200635e-14
Exon 19-20	5976894	5977811	-1.026052986	1.626187e-10	8.866688e-08

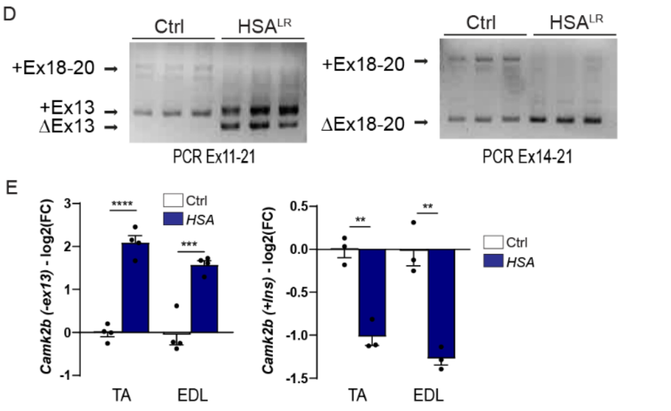

F

Ctrl = 100 %	HSA^LR			Mbnl1	Patient
Camk2b	GA	TA	EDL	TA	TA
Exclusion Exon 13	420%	434%	300%	517%	✓
Inclusion Exon 18	47%			31%	✓
Inclusion Exon 19	46%	50%	42%	29%	✓
Inclusion Exon 20	44%			26%	✓

Figure S2, related to Figure 2: Mis-splicing of *Camk2b* genes in *HSA^{LR}* muscle. A Alternative splicing of *Camk2b* transcript, with the corresponding encoded protein domains of CaMKIIβ. **B, C** RNA-seq results for *Camk2b* showing mis-spliced regions in *HSA^{LR} gastrocnemius* muscle, compared to control. n=4. Statistical analysis is given in C, with the log2FC corresponding to the log-fold change in the expression of one exon, normalized to the expression of all the exons of the same gene, in *HSA^{LR}* muscle, as compared to controls. **D** Splicing analysis of exon 13 and exons 18-20 of *Camk2b* by end-point PCR. The scheme of the gene and the primers used are given in A. **E, F** Quantitative RT-PCR of exon 13 exclusion and exons 18-20 inclusion in TA and EDL muscles from *HSA^{LR}* mice. Data are normalized on levels of total *Camk2b* transcripts, relative

to control and expressed as log2(Fold Change). n = 4 (Ex13) and 3 (+Ins) per group. A summary table of *Camk2b* mis-splicing is given in F. All data are mean ± SEM; ** $p<0.01$; *** $p<0.0001$; **** $p<0.0001$; two-tailed unpaired Student's t-test.

Figure S3

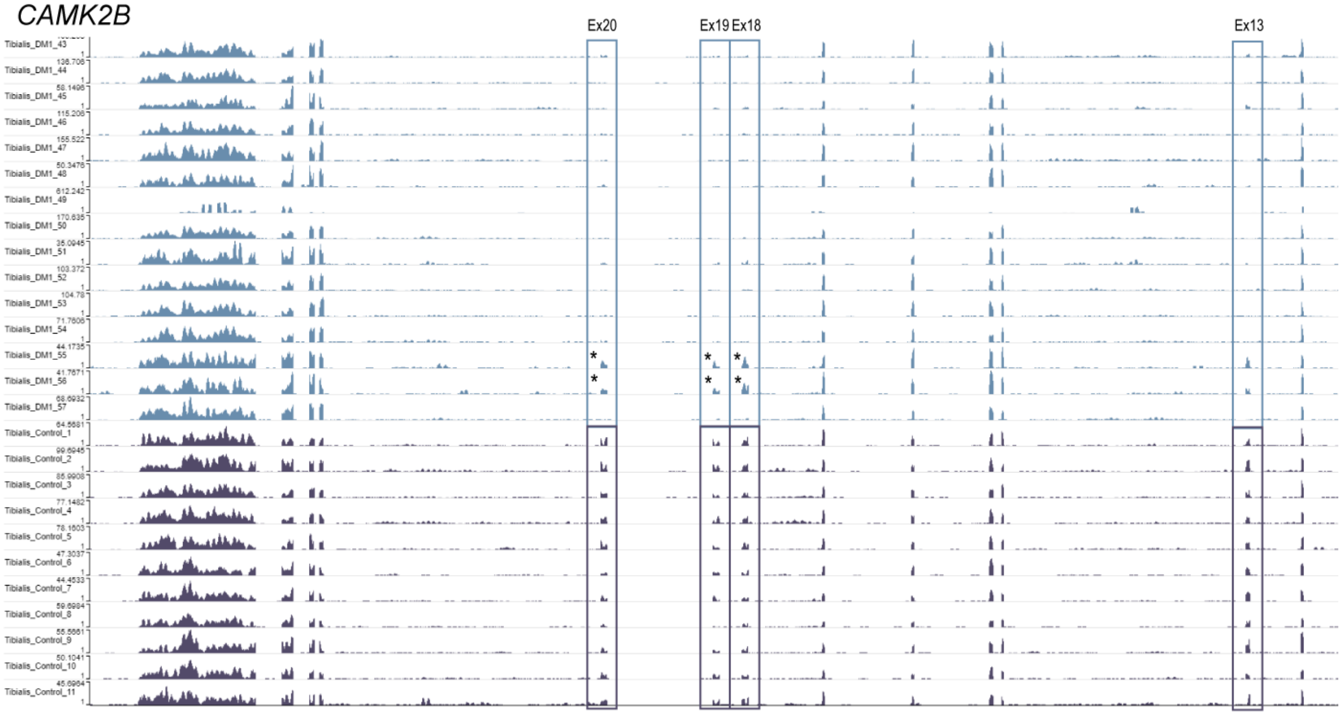

Figure S3, related to Figure 2: Mis-splicing of *CAMK2B* gene in muscle from DM1 patients. RNA-seq results showing mis-splicing of *CAMK2B* exons 13, 18, 19 and 20 in TA muscle from DM1 patients (blue), compared to control individuals (purple). Data are from *dmseq.org* [5]. Asterisks show two DM1 muscles with normal splicing of *CAMK2B*.

Figure S4

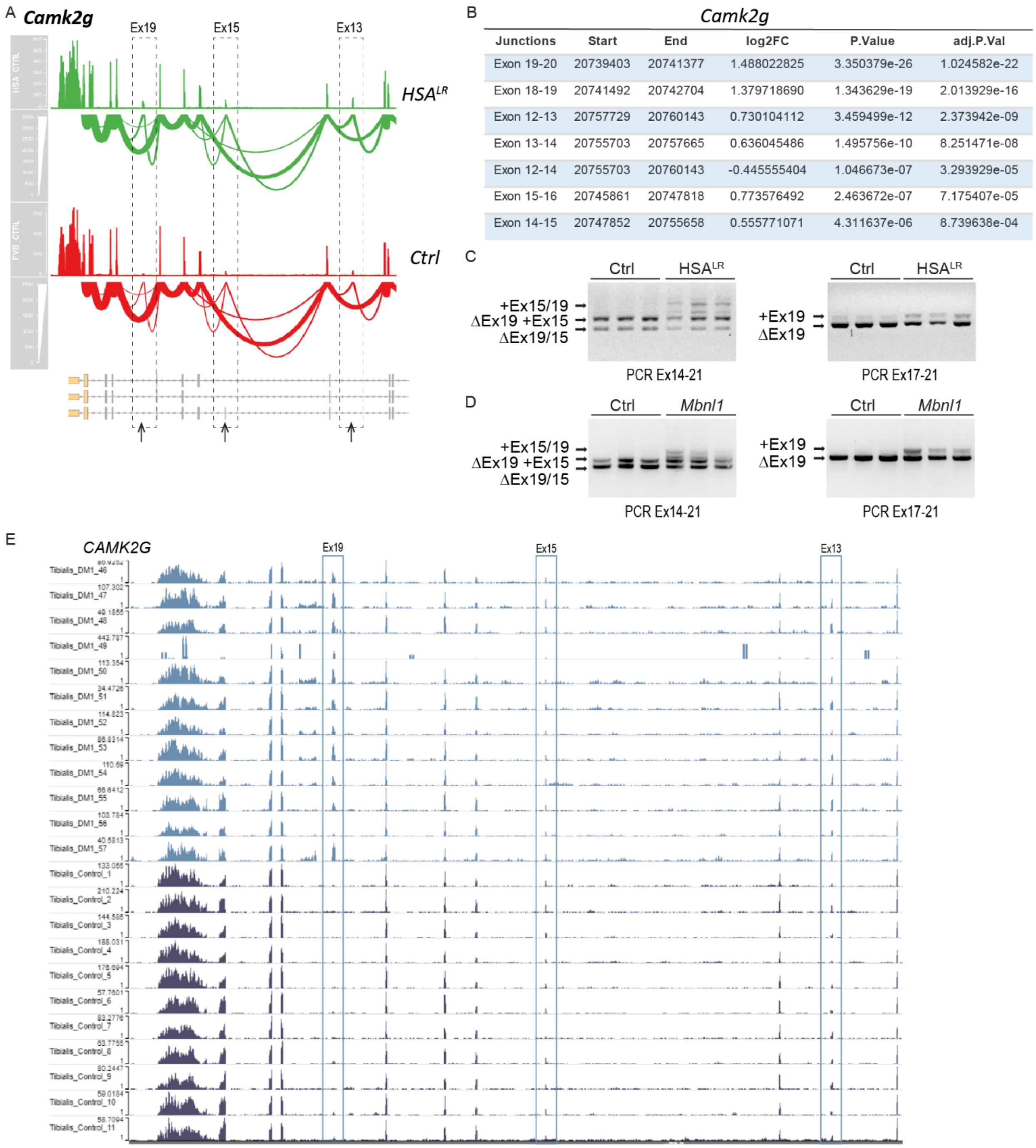

Junctions	Start	End	log2FC	P.Value	adj.P.Val
Exon 19-20	20739403	20741377	1.488022825	3.350379e-26	1.024582e-22
Exon 18-19	20741492	20742704	1.379718690	1.343629e-19	2.013929e-16
Exon 12-13	20757729	20760143	0.730104112	3.459499e-12	2.373942e-09
Exon 13-14	20755703	20757665	0.636045486	1.495756e-10	8.251471e-08
Exon 12-14	20755703	20760143	-0.445555404	1.046673e-07	3.293929e-05
Exon 15-16	20745861	20747818	0.773576492	2.463672e-07	7.175407e-05
Exon 14-15	20747852	20755658	0.555771071	4.311637e-06	8.739638e-04

Figure S4, related to Figure 2: Mis-splicing of *Camk2g* genes in DM1 muscle. A, B RNA-seq results for *Camk2g* showing the mis-spliced regions in *HSA*^{LR} muscle, compared to control. Statistical analysis is given in B, with the log2FC corresponding to the log-fold change in the expression of one exon, normalized to

the expression of all the exons of the same gene, in *HSA*^{LR} muscle, as compared to controls. n = 4 per group. **C, D** End-point PCR analysis of mis-splicing of exons 15 and 19 of *Camk2g* in *gastrocnemius* from *HSA*^{LR} *mice* (C) and TA muscle from *Mbnl1*$^{\Delta E3/\Delta 3E}$ mice (D). n=3 per group. **E** RNA-seq results for *CAMK2G* showing mis-splicing of exon 19 in TA muscle from DM1 patients (blue), compared to control individuals (purple). Data are from *dmseq.org* [5].

Figure S5

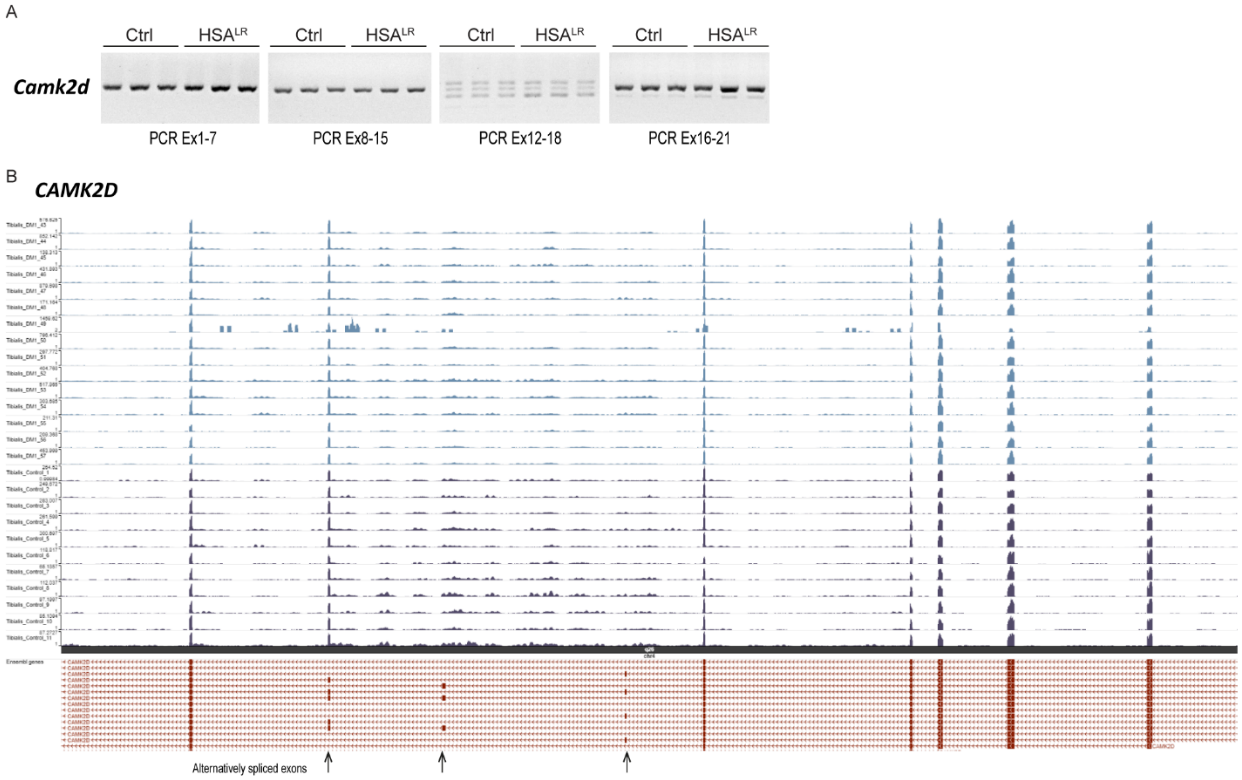

Figure S5, related to Figure 2: Splicing of *CAMK2D* gene in muscle from *HSA^{LR}* mice and DM1 patients. A End-point PCR analysis of exons 1 to 21 splicing of *Camk2d* in *gastrocnemius* from *HSA^{LR}* and control muscles. n=3 per group. **B** RNA-seq results for *CAMK2D* showing normal splicing of 3 predicted alternative exons in TA muscle from DM1 patients, compared to control individuals. Data are from *dmseq.org* [5].

Figure S6

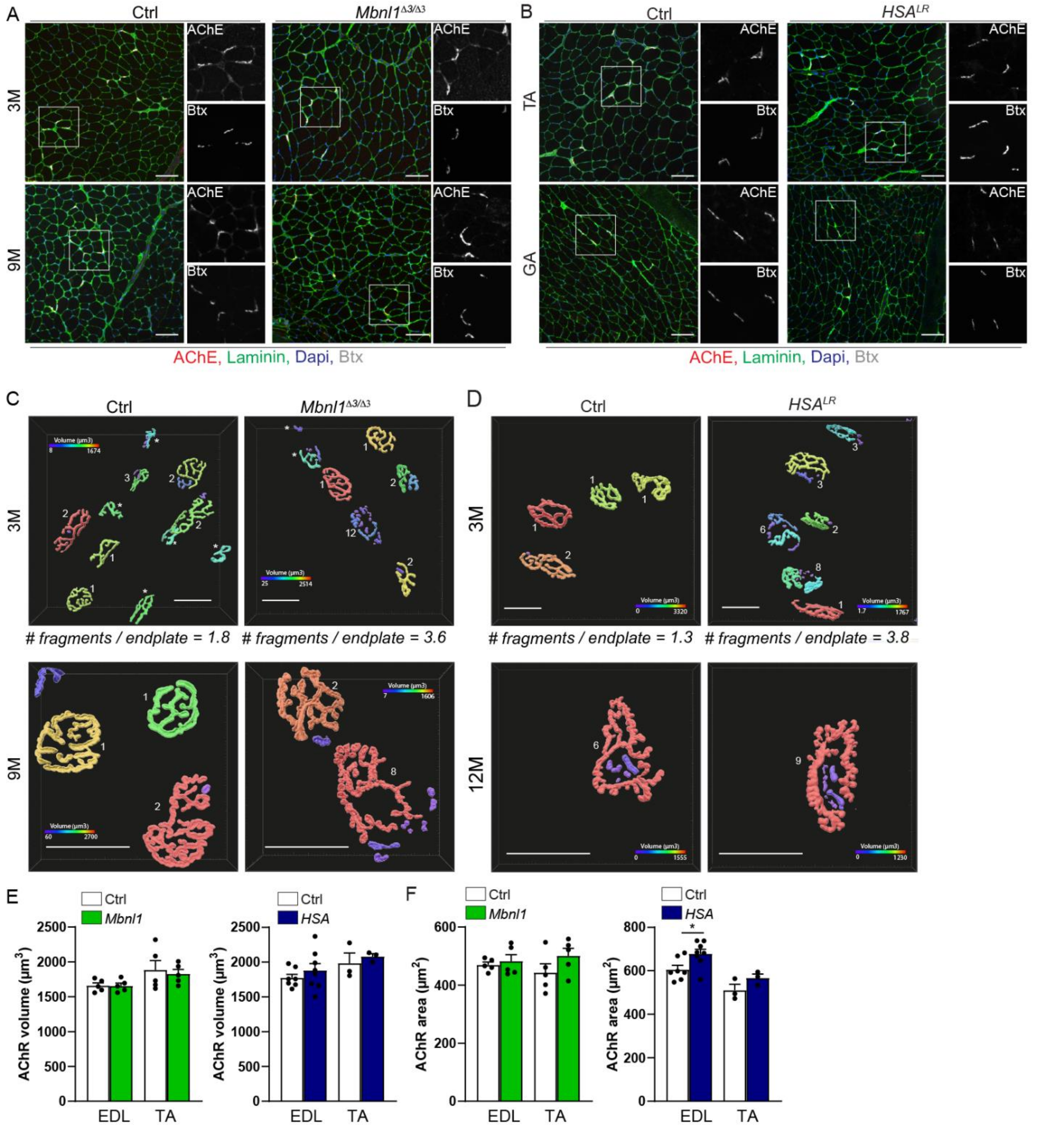

Figure S6, related to Figure 3: Endplate perturbations in *Mbnl1*$^{\Delta E3/\Delta E3}$ and *HSA*^{LR} muscles. A, B Fluorescent images of TA or *gastrocnemius* (GA) muscles stained with antibodies against acetylcholinesterase (AChE, red) and laminin (green), α-bungarotoxin (Btx; grey) and Dapi (blue). Muscles are from 3- or 9-month(M)-

old *Mbnl1*$^{\Delta E3/\Delta E3}$ (A) and 3-month-old *HSA*^{LR} (B) mice. Scale bar, 100 µm. Higher magnification panels show NMJ regions positive for Btx and acetylcholinesterase. **C, D** 3D reconstructed image of Btx-stained endplates shown in Fig. 3A, B. The colours are determined by the volume of the AChR fragments. The number of fragments per endplate, as well as the mean number of fragments per endplate for each field, are indicated. Asterisks show incomplete endplates. Scale bar, 50 µm. **E, F** Volume (E) and projected area (F) of AChRs per endplate in EDL and TA muscles from 3-month-old *Mbnl1*$^{\Delta E3/\Delta E3}$ and *HSA*^{LR} mice. n=5 Ctrl/*Mbnl1*$^{\Delta E3/\Delta E3}$; 7/8 EDL and 3TA Ctrl/*HSA*^{LR}. All data are mean ± SEM; * $p<0.05$; two-tailed unpaired Student's t-test.

Figure S7

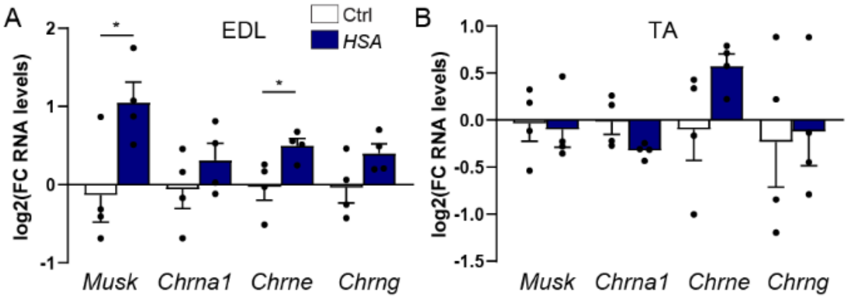

Figure S7, related to Figure 5: Changes in synaptic gene expression in *Mbnl1$^{ΔE3/ΔE3}$* and *HSA^{LR}* muscles. Quantitative PCR analysis of *Musk, Chrna1, Chrne* and *Chrng* in EDL (A) and TA (B) muscles from 3-month-old *HSA^{LR}* mice. Levels are normalized to *Tbp,* relative to control and expressed as log2(Fold Change). n=4 per group. All data are mean ± SEM; * $p<0.05$; two-tailed unpaired Student's t-test.

Figure S8

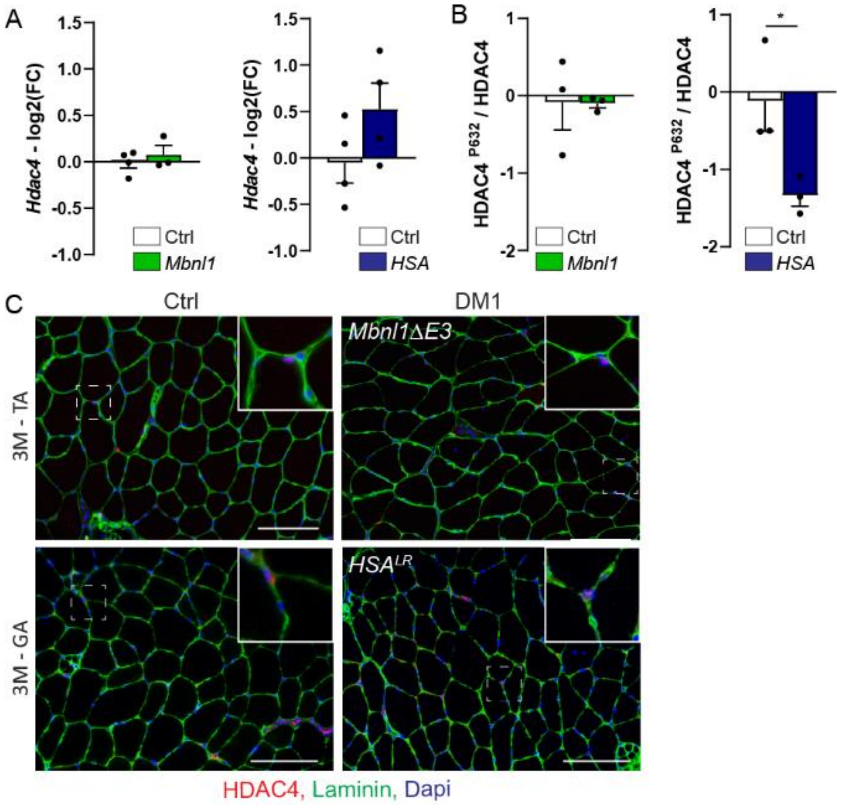

Figure S8, related to Figure 6: Changes in HDAC4 downstream targets in *Mbnl1*$^{\Delta E3/\Delta E3}$ and *HSA*^{LR} muscles.

A mRNA levels of *Hdac4* in *Mbnl1*$^{\Delta E3/\Delta E3}$ and *HSA*^{LR} muscles. RNA levels are normalized to *Tbp* expression, relative to control muscle and expressed as log2(Fold Change). n = 4/3 Ctrl/*Mbnl1*$^{\Delta E3/\Delta E3}$ and 4/4 Ctrl/*HSA*^{LR}. **B** Levels of phosphorylated HDAC4 (Ser632) in *Mbnl1*$^{\Delta E3/\Delta E3}$ and *HSA*^{LR} muscles. Protein levels are normalized to total HDAC4 levels, relative to control muscle and expressed as log2(Fold Change). n = 3 per group. **C** Fluorescent image of *Mbnl1*$^{\Delta E3/\Delta E3}$ and *HSA*^{LR} muscles stained with antibodies against HDAC4 (red), laminin (green) and Dapi (blue). Scale bar, 100 µm. Higher magnification panels show positive myonuclei. All data are mean ± SEM; * $p<0.05$; two-tailed unpaired Student's t-test.

Figure S9

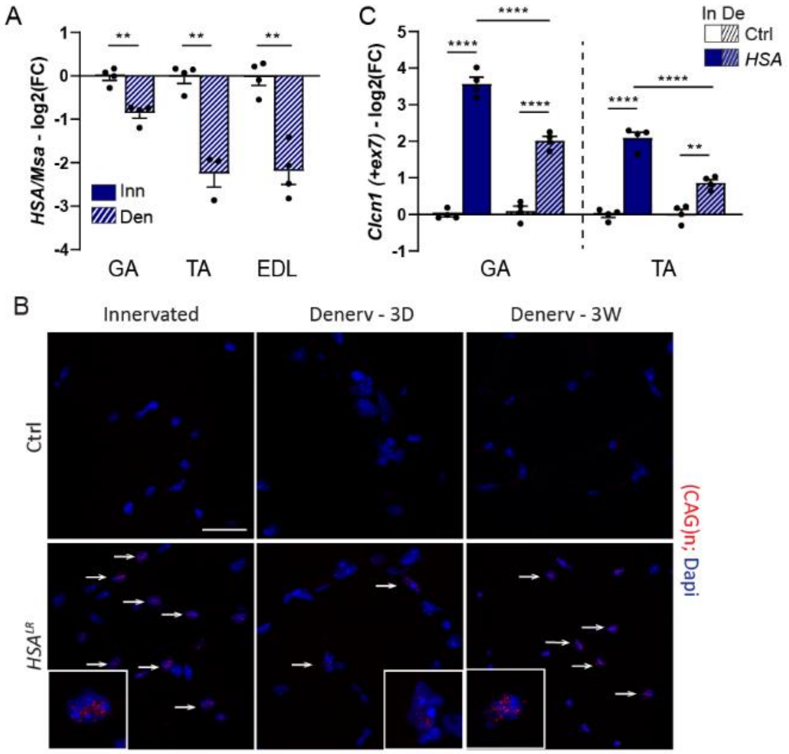

Figure S9, related to Figure 7: Limits of the *HSA*^{LR} mouse model upon nerve injury. A Quantitative PCR analysis of human *ACTA1* in *gastrocnemius,* TA and EDL in *HSA*^{LR} innervated muscle and after 3 days of denervation. Results are normalized on mouse *Acta1*, relative to innervated *HSA*^{LR} muscle and expressed as log2(Fold Change). n=4 per group. **B** Fluorescent *in situ* hybridization with Cy3-CAG$_{10}$ DNA probe, on sections of innervated and denervated (3 days (D) and 3 weeks (W)) muscles from control and *HSA*^{LR} mice. Arrows point to positive nuclei. Scale bar, 25 µm. **C** Quantitative PCR analysis of spliced transcripts of *Clcn1* in TA and *gastrocnemius* muscles from control and *HSA*^{LR} mice after 3 days of denervation. Levels are normalized on total transcript levels, relative to innervated control muscle and expressed as log2(Fold Change). n=4 per group. Data are mean ± SEM; ** p<0.01; ****p<0.0001; two-tailed unpaired Student's t-test (A) and two-way ANOVA with a Tukey's post-hoc analysis (C).

Figure S10

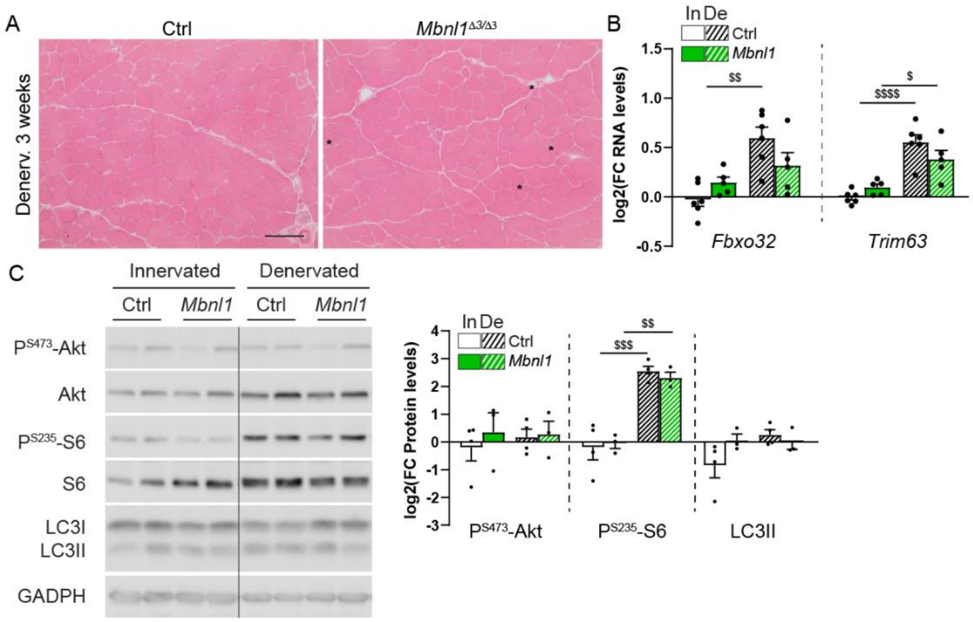

Figure S10, related to Figure 7: Muscle response to denervation in *Mbnl1*$^{\Delta E3/\Delta E3}$ mice. A H&E coloration shows no major alterations in denervated muscle from *Mbnl1*$^{\Delta E3/\Delta E3}$ and control mice, 3 weeks after nerve injury. Scale bar, 100 µm. **B** Quantitative PCR analysis of *Fbxo32* and *Trim63* in innervated and 3-day-denervated muscles from *Mbnl1*$^{\Delta E3/\Delta E3}$ mice. Transcript levels are normalized to *Tbp,* relative to control innervated muscle and expressed as log2(Fold Change). n=6/5 Ctrl/*Mbnl1*$^{\Delta E3/\Delta E3}$. **C** Western blot analysis of Akt/mTORC1 pathway in total protein lysate of TA muscle from *Mbnl1*$^{\Delta E3/\Delta E3}$ mice. Quantification is given for Akt and S6 phosphorylated levels and LC3II levels. Levels are normalized to GAPDH, relative to control innervated muscle and expressed as log2(Fold Change). n = 4/3 Ctrl/*Mbnl1*$^{\Delta E3/\Delta E3}$. All data are mean ± SEM; $^{\$}$ $p<0.05$; $^{\$\$}$ $p<0.01$; $^{\$\$\$}$ $p<0.001$; $^{\$\$\$\$}$ $p<0.0001$ between Inn/Den; two-way ANOVA with a Tukey's post-hoc analysis (Inn/Den and Ctrl/ *Mbnl1*$^{\Delta E3/\Delta E3}$).

Figure S11

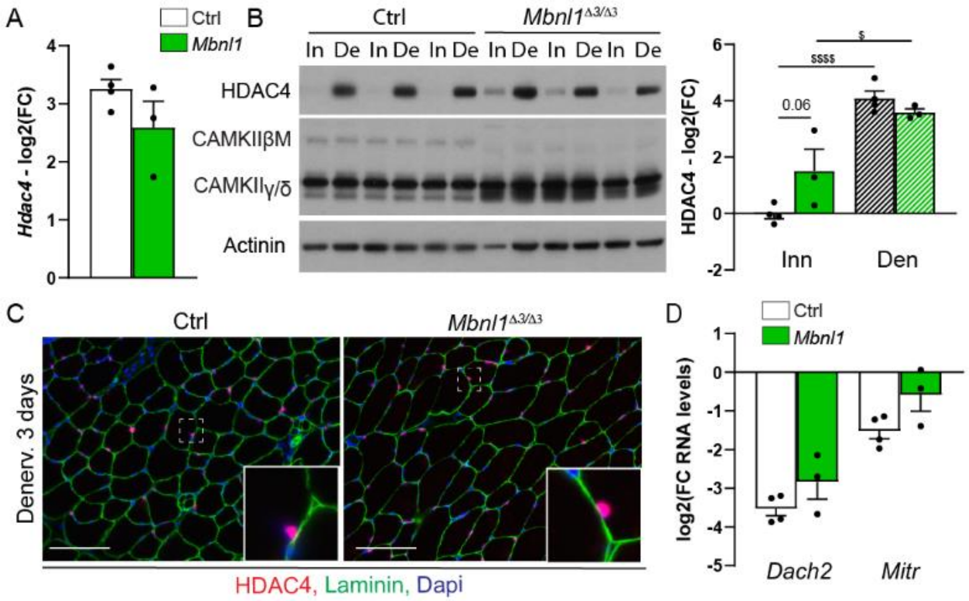

Figure S11, related to Figure 8. HDAC4 is efficiently induced in denervated *Mbnl1*$^{\Delta E3/\Delta E3}$ muscle. A Quantitative RT-PCR of *Hdac4* transcript levels in denervated (3 days) muscles from control and *Mbnl1*$^{\Delta E3/\Delta E3}$ mice. Transcript levels are normalized to *Tbp*, relative to innervated control and expressed as log2(Fold Change). n = 4/3 Ctrl/*Mbnl1*$^{\Delta E3/\Delta E3}$. **B** Western blot analysis of HDAC4 protein levels in innervated (In) and denervated (De, 3 days) muscles from control and *Mbnl1*$^{\Delta E3/\Delta E3}$ mice. Protein levels are normalized to α-actinin. Levels are relative to innervated control and expressed as log2(Fold Change). n = 4/3 Ctrl/*Mbnl1*$^{\Delta E3/\Delta E3}$. **C** Fluorescent image of *Mbnl1*$^{\Delta E3/\Delta E3}$ denervated muscle stained with antibodies against HDAC4 (red), laminin (green) and Dapi (blue). Scale bar, 100 µm. Higher magnification panels show HDAC4-positive myonuclei. **D** Quantitative RT-PCR analysis of *Dach2* and *Mitr* in TA muscle from control and *Mbnl1*$^{\Delta E3/\Delta E3}$ mice 3 days post-denervation. Levels are normalized to *Tbp,* relative to control innervated and expressed as log2(Fold Change). n = 4/3 Ctrl/*Mbnl1*$^{\Delta E3/\Delta E3}$. All data are mean ± SEM; $^{\$}$ $p<0.05$, $^{\$\$\$\$}$ $p<0.0001$ between Inn/Den; two-way ANOVA with a Tukey's post-hoc analysis (Inn/Den and Ctrl/ *Mbnl1*$^{\Delta E3/\Delta E3}$).

Figure S12

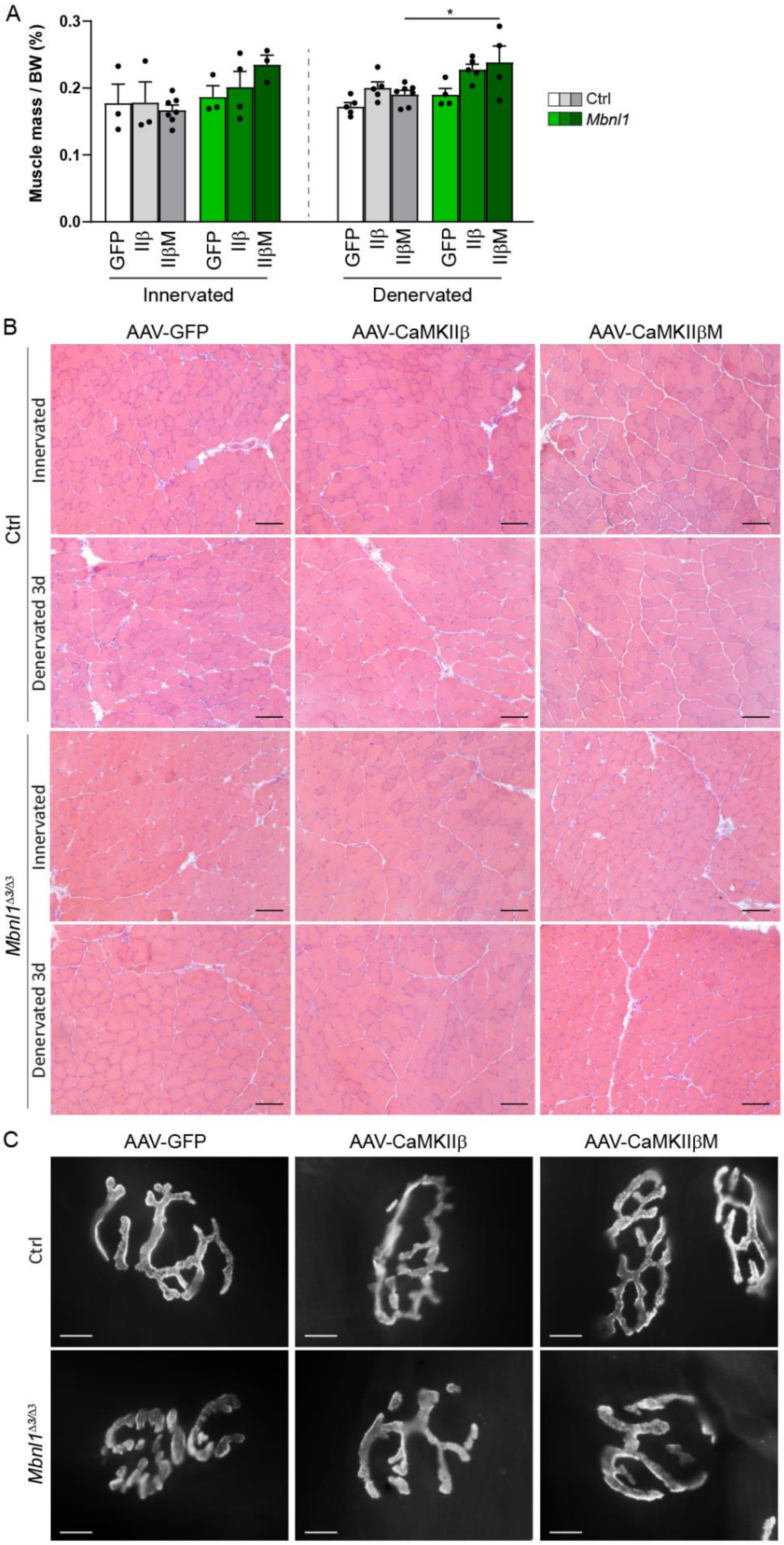

A
Muscle mass / BW (%)
0.3
0.2
0.1
0.0
*
GFP
IIβ
IIβM
GFP
IIβ
IIβM
Innervated
GFP
IIβ
IIβM
GFP
IIβ
IIβM
Denervated
Ctrl
Mbnl1
B
AAV-GFP
AAV-CaMKIIβ
AAV-CaMKIIβM
Innervated
Denervated 3d
Ctrl
Innervated
Denervated 3d
Mbnl1Δ3/Δ3
C
AAV-GFP
AAV-CaMKIIβ
AAV-CaMKIIβM
Ctrl
Mbnl1Δ3/Δ3

Figure S12, related to Figure 9: CaMKIIβ overexpression does not perturb muscle phenotype in *Mbnl1*$^{\Delta E3/\Delta E3}$ mice. A Mass of control and *Mbnl1*$^{\Delta E3/\Delta E3}$ TA innervated and denervated muscles infected with AAV-GFP, -CaMKIIβ, or -CaMKIIβM. Masses are normalized to total body weight. n = 3/3/7 Ctrl Inn, 5/5/7 Ctrl Den, 3 *Mbnl1*$^{\Delta E3/\Delta E3}$ per group (except for Den IIβM, n=4). All data are mean ± SEM. **B** H&E coloration reveals no major alterations in control and *Mbnl1*$^{\Delta E3/\Delta E3}$ TA innervated and denervated (3 days) muscles infected with AAV-GFP, -CaMKIIβ, or -CaMKIIβM. Scale bar, 200 µm. **C** Fluorescent images of endplates stained with α-bungarotoxin in EDL muscles from control and *Mbnl1*$^{\Delta E3/\Delta E3}$ mice injected with AAV-GFP, -CaMKIIβ, or -CaMKIIβM. Scale bar, 10 µm.

Figure S13

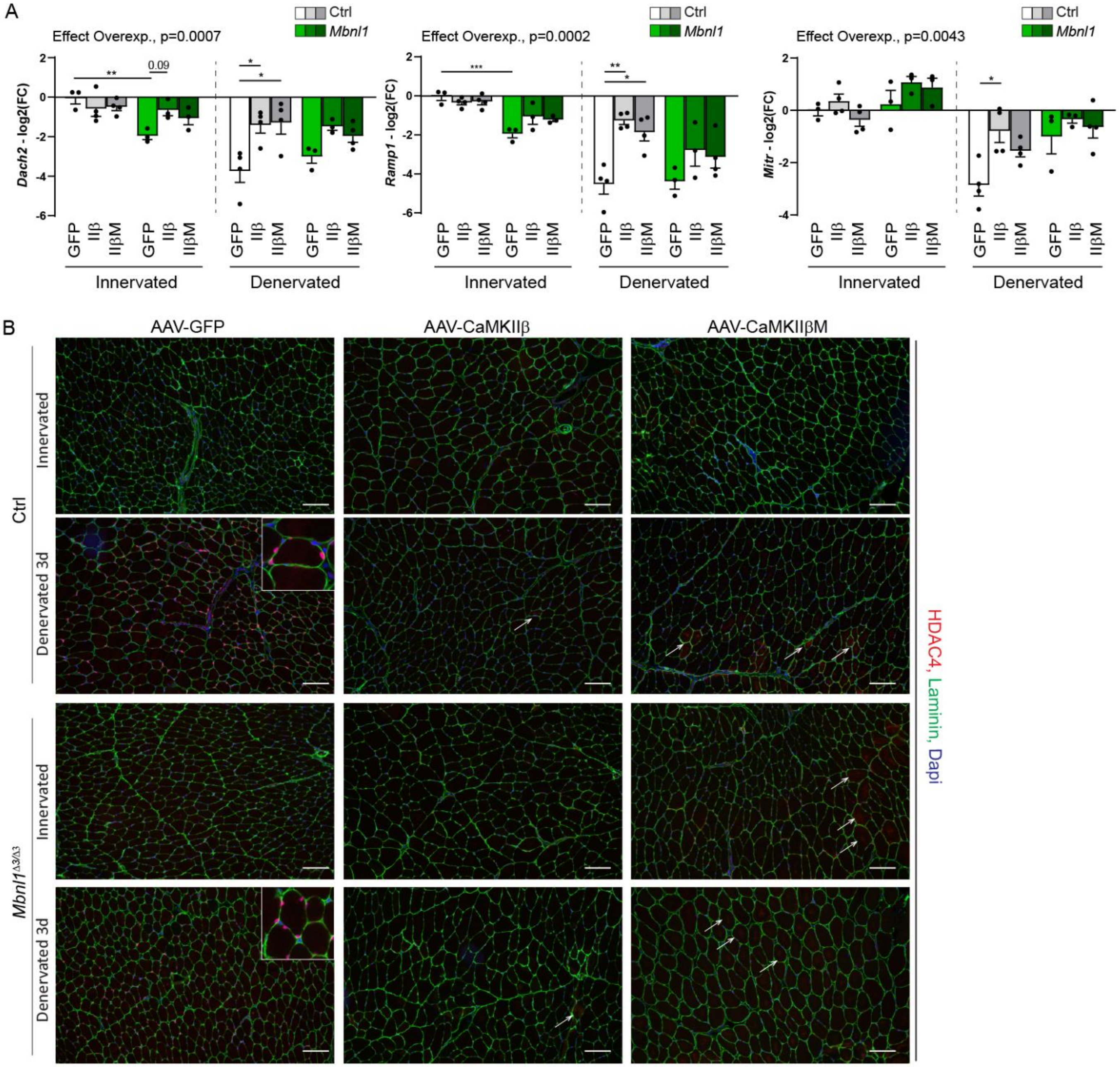

Figure S13, related to Figure 9: CaMKIIβ overexpression inhibits HDAC4 pathway in control and mutant muscles. A mRNA levels of *Dach2, Ramp1* and *Mitr* in control and *Mbnl1*$^{\Delta E3/\Delta E3}$ TA innervated and denervated (3 days) muscles injected with AAV-GFP, -CaMKIIβ, or -CaMKIIβM. Levels are normalized to *Tbp,* relative to control innervated muscle and expressed as log2(Fold Change). n = 4 Ctrl (except for Inn GPF, n=3) and 3 *Mbnl1*$^{\Delta E3/\Delta E3}$ (except for Den IIβM, n=4). Data are mean ± SEM; *p<0.05; ** p<0.01; two-

way ANOVA with Tukey's post-hoc correction. **B** Fluorescent images of control and *Mbnl1*$^{\Delta E3/\Delta E3}$ innervated and denervated (3 days) muscles stained with antibodies against HDAC4 (red), laminin (green) and Dapi (blue). Scale bar, 100 µm. Higher magnification panels show HDAC4-positive myonuclei. Arrows point to HDAC4 cytoplasmic accumulation.

Table S1: List of primers

Gene	Forward primer	Reverse primer
Acta1 (mouse)	5'-CCGGAAAGAAATCTCAACCA-3'	5'-CCAAGTCCTGCAAGTGAACA-3'
ACTA1 (human)	5'-CGAGACCACCTACAACAGCA-3'	5'-GGCATACAGGTCCTTCCTGA-3'
Actc1	5’-TTTGTCACCACTGCTGAACG-3’	5’-GAGAGGAGGAAGATGCAGCT-3’
Atp2a1+ex22	5'- GCCCTGGACTTTACCCAGTG-3'	5'-ACGGTTCAAAGACATGGAGGA-3'
Atp2a1 pan	5'- GCCCTGGACTTTACCCAGTG-3'	5'-CCTCCAGATAGTTCCGAGCA-3'
Camk2b-ex13	5'-TTTCTCAGCAGCCAAGAGTTT-3'	5'-TTCCTTAATCCCGTCCACTG-3'
Camk2b pan	5'-GCACGTCATTGGCGAGGA-3'	5'-ACGGGTCTCTTCGGACTGG-3'
Camk2b-ex18	5'-CCTGATGTCCTGAGCTTGGT-3'	5'-GAACTGGAGATTGGCAGGAG-3’
Camk2b-ex19	5'-TCAGTGAGAAGGGGCTGTG-3'	5'-CTAGGAGACCCGGAGACAAG-3’
Camk2b-ex20	5'-CCCCCAGGATCTCTGACA-3'	5'-TGCTTCCGGGATGGGGTGGGC-3’
Camk2b-ex13 (gel)	5'-GTTCCACCGTGGCCTCTAT-3'	5'-TCGGAAGATTCCAGGGCAGC-3’
Camk2b-ex18-20 (gel)	5'-CCAGACAAACAGCACCAAAA-3'	5'-TGAGCTGCTCTGTGGTCTTG-3’
Camk2g+exon 15/19 (gel)	5'-AGTTCCAGC GTGCACCTAAT-3'	5'-ACGTGGACGTGAGGGTTTAG-3’
Camk2g+exon 19 (gel)	5'-ACACCACTACAGAAGACGAAGA-3'	5'-AACCTCAAACGAACAGGACC-3’
Cdh1	5’-TTTAAGCCCAGCACTCAGGA-3’	5’-GGGTTTCTCTGTGTAGCCCT-3’
Clcn1+ex7a	5'-GGGCGTGGGATGCTACTTTG-3'	5'- AGGACACGGAACACAAAGGC-3'
Clcn1 pan	5'-CTGACATCCTGACAGTGGGC-3'	5'- AGGACACGGAACACAAAGGC-3'
Chrna1	5'-TCCCTTCGATGAGCAGAACT-3'	5'-GGGCAGCAGGAGTAGAACAC-3'
Chrng	5'-GTGTCTTCGAGGTGGCTCTC-3'	5'-TCTGGGATTGGAAGATGAGG-3'
Dach2	5'-CCAGCTCAAATCCCAGTCAT-3'	5'-CGCAGTTCCTTCTTTTCCTG-3'
Dhrs7c	5’-AGGACTGGGAAAGGAGTGTG-3’	5’-TGGTCAAGGTGGCATAGAGG-3’
Hdac4	5'-CAGACAGCAAGCCCTCCTAC-3'	5'-AGACCTGTGGTGAACCTTGG-3'
Mitr	5'-CCTGCAGCACCTACTGTTGA-3'	5'-GTACCTCTAATGCCCGGTGA-3'
Myogenin	5'-ACTCCCTTACGTCCATCGTG-3'	5'-CAGGACAGCCCCACTTAAAA-3'
Myh2	5’-ACAAATCTATCCAAGTTCCG-3'	5'-TTCGGTCATTCCACAGCATC-3'
Myh4	5’-CAGATGAAAAGGTGGCCATT-3'	5'-CTTCCCTTTGCTTTTGCTTG-3'
Ramp1	5’-GGAAGGGTGGGGAAGAATGA-3’	5’-TCCATGTCAAAGTCCGGGAA-3’
Tbp	5'-CTCAGTTACAGGTGGCAGCA-3'	5'-CAGCACAGAGCAAGCAACTC-3'
